# Discriminant Analysis under *f*-Divergence Measures

**DOI:** 10.3390/e24020188

**Published:** 2022-01-27

**Authors:** Anmol Dwivedi, Sihui Wang, Ali Tajer

**Affiliations:** Department of Electrical, Computer, and Systems Engineering, Rensselaer Polytechnic Institute, Troy, NY 12180, USA; dwivea2@rpi.edu (A.D.); scottwon@bupt.edu.cn (S.W.)

**Keywords:** dimensionality reduction, discriminant analysis, *f*-divergence, statistical inference

## Abstract

In statistical inference, the information-theoretic performance limits can often be expressed in terms of a statistical divergence between the underlying statistical models (e.g., in binary hypothesis testing, the error probability is related to the total variation distance between the statistical models). As the data dimension grows, computing the statistics involved in decision-making and the attendant performance limits (divergence measures) face complexity and stability challenges. Dimensionality reduction addresses these challenges at the expense of compromising the performance (the divergence reduces by the data-processing inequality). This paper considers linear dimensionality reduction such that the divergence between the models is maximally preserved. Specifically, this paper focuses on Gaussian models where we investigate discriminant analysis under five *f*-divergence measures (Kullback–Leibler, symmetrized Kullback–Leibler, Hellinger, total variation, and $\chi^{2}$). We characterize the optimal design of the linear transformation of the data onto a lower-dimensional subspace for zero-mean Gaussian models and employ numerical algorithms to find the design for general Gaussian models with non-zero means. There are two key observations for zero-mean Gaussian models. First, projections are not necessarily along the largest modes of the covariance matrix of the data, and, in some situations, they can even be along the smallest modes. Secondly, under specific regimes, the optimal design of subspace projection is identical under all the *f*-divergence measures considered, rendering a degree of universality to the design, independent of the inference problem of interest.

## 1. Introduction

### 1.1. Motivation

Consider a simple binary hypothesis testing problem in which we observe an *n*-dimensional sample *X* and aim to discern the underlying model according to:

(1) $$\begin{array}{c} H_{0}:\;X∼P\;\mathrm{vs}.\;H_{1}:\;X∼Q. \end{array}$$

The optimal decision rule (in the Neyman-Pearson sense) involves computing the likelihood ratio $\frac{dP}{dQ}\left(X\right)$ and the performance limit (sum of type I and type II errors) is related to the total variation distance between P and Q. We emphasize that our focus is on the settings in which the *n* elements of *X* are not statistically independent, in which case the likelihood ratio $\frac{dP}{dQ}\left(X\right)$ cannot be decomposed into the product of the coordinate-level likelihood ratios. One of the key practical obstacles to solve such problems pertains to the computational cost of finding and performing the statistical tests. This renders a gap between the performance that is information-theoretically viable (unbounded complexity) versus a performance possible under bounded computational complexity [1,2].

Dimensionality reduction techniques have become an integral part of statistical analysis in high dimensions [3,4,5,6]. In particular, linear dimensionality reduction methods have been developed and used for over a century for various reasons, such as their low computational complexity and simple geometric interpretation, as well as for a multitude of applications, such as data compression, storage, and visualization, to name only a few. These methods linearly map the high-dimensional data to lower dimensions while ensuring that the desired features of the data are preserved. There exist two broad sets of approaches to linear dimensionality reduction in one dataset *X*, which we review next.

### 1.2. Related Literature

*(1)**Feature extraction**:* In one set of approaches, the objective is to select and extract informative and non-redundant features in the dataset *X*. These approaches are generally unsupervised. These widely-used approaches are principal component analysis (PCA), and its variations [7,8,9], multidimensional scaling (MDS) [10,11,12,13], and sufficient dimensionality reduction (SDR) [14]. The objective of PCA is to retain as much variation in the data in a lower dimension by minimizing the reconstruction error. In contrast, MDS aims to maximize the scatter of the projection and maximizes an aggregate scatter metric. Finally, the objective of SDR is to design an orthogonal mapping of the data that makes the data *X* and the responses conditionally independent (given the projected data). There exist extensive variations to the three approaches, and we refer the reader to Reference [6] for more discussions.

*(2)**Class separation**:* In another set of approaches, the objective is to perform classification in the lower dimensional space. These approaches are supervised. Depending on the problem formulation and the underlying assumptions, the resulting decision boundaries between the models can be linear or non-linear. One approach pertinent to this paper’s scope is discriminant analysis (DA), that leverages the distinction between given models and designs a mapping such that its lower-dimensional output exhibits maximum separation across different models [15,16,17,18,19,20]. In general, this approach generates two matrices: within-class and between-class scatter matrices. The within-class scatter matrix shows the scatter of the samples around their respective class means, whereas, in contrast, the between-class scatter matrix captures the scatter of the samples around the mixture mean of all the models. Subsequently, a univariate function of these matrices is formed such that it increases when the between-class scatter becomes larger, or when the within-class scatter becomes smaller. Examples of such a function of between-class and within-class matrices is a classification index that includes the ratio of their determinants, difference of their determinants, and ratio of their traces [17]. These approaches focus on reducing the dimension to one and maximize separability between the two classes. There exist, however, studies that consider reducing to dimensions higher than one and separation across more than two classes. Finally, depending on the structure of the class-conditional densities, the resulting shape of the decision boundaries give rise to linear and quadratic DA.

The *f*-divergences between a pair of probability measures quantifies the similarity between them. Shannon [21] introduced the mutual information as a divergence measure, which was later studied comprehensively by Kullback and Leibler [22] and Kolmogorov [23], establishing the importance of such measures in information theory, probability theory, and related disciplines. The family of *f*-divergences, independently introduced by Csiszár [24], Ali and Silvey [25], and Morimoto [26], generalize the Kullback–Leibler divergence which enable characterizing the information-theoretic performance limits of a wide range of inference, learning, source coding, and channel coding problems. For instance, References [27,28,29,30] consider their application to various statistical decision-making problems [31,32,33,34]. More recent developments on the properties of *f*-divergence measures can be found in References [31,35,36,37].

### 1.3. Contributions

The contribution of this paper has two main distinctions from the existing literature on DA. First, DA generally focuses on the classification problem for determining the underlying model of the data. Secondly, motivated by the complexities of finding the optimal decision rules for classification (e.g., density estimation), the existing criteria used for separation are selected heuristically. In this paper, we study this problem by referring to the family of *f*-divergences as measures of the distinction between a pair of probability distributions. Such a choice has three main features: (i) it enables designing linear mappings for a wider range of inference problems (beyond classification); (ii) it provides the designs that are optimal for the inference problem at hand; and (iii) it enables characterizing the information-theoretic performance limits after linear mapping. Our analyses are focused on Gaussian models. Even though we observe that the design of the linear mapping has differences under different *f*-divergence measures, we have two main observations in the case of zero-mean Gaussian models: (i) the optimal design of the linear mapping is not necessarily along the most dominant components of the data matrix; and (ii) in certain regimes, irrespective of the choice of the *f*-divergence measure, the design of the linear map that retains the maximal divergence between the two models is robust. In such cases, this makes the optimal design of the linear map independent of the inference problem at hand rendering a degree of universality (in the considered space of the Gaussian probability measures).

The remainder of the paper is organized as follows. Section 2 provides the linear dimensionality reduction model, and it provides an overview of the *f*-divergence measures considered in this paper. Section 3 formulates the problem, and it helps to facilitate the mathematical analysis in subsequent sections. In Section 4, we provide a motivating operational interpretation for each *f*-divergence measure and then characterize an optimal design of the linear mapping for zero-mean Gaussian models. Section 5 considers numerical simulations for inference problems associated with the *f*-divergence measure of interest for zero-mean Gaussian models. Section 6 generalizes the theory to non-zero mean Gaussian models and discusses numerical algorithms that help characterize the design of the linear map, and Section 7 concludes the paper. A list of abbreviations used in this paper is provided on page 22.

## 2. Preliminaries

Consider a pair of *n*-dimensional Gaussian models:

(2) $$\begin{array}{c} P:\;\;N\left(\mu_{P},\Sigma_{P}\right),\;\mathrm{and}\;Q:\;\;N\left(\mu_{Q},\Sigma_{Q}\right), \end{array}$$

where $\mu_{P},\mu_{Q}$ and $\Sigma_{P},\Sigma_{Q}$ are two distinct mean vectors and covariance matrices, respectively, and P and Q denote their associated probability measures. The nature selects one model and generates a random variable $X\in R^{n}$. We perform linear dimensionality reduction on *X* via matrix $A\in R^{r\times n}$, where r<n, rendering

(3) $$\begin{array}{c} Y\;\overset{▵}{=}A\cdot X. \end{array}$$

After linear mapping, the two possible distributions of *Y* induced by matrix A are denoted by $P_{A}$ and $Q_{A}$, where

(4) $$\begin{array}{c} \begin{array}{cc} P_{A}\;: & N(A\cdot \mu_{P},A\cdot \Sigma_{P}\cdot A^{⊤})\\ Q_{A}: & N(A\cdot \mu_{Q},A\cdot \Sigma_{Q}\cdot A^{⊤}) \end{array}. \end{array}$$

Motivated by inference problems that we discuss in Section 3, our objective is to design the linear mapping parameterized by matrix A that ensures that the two possible distributions of *Y*, i.e., $P_{A}$ and $Q_{A}$, are maximally distinguishable. That is, to design A as a function of the statistical models (i.e., $\mu_{P}$, $\mu_{Q}$, $\Sigma_{P}$ and $\Sigma_{Q}$) such that relevant notions of *f*-divergences between $P_{A}$ and $Q_{A}$ are maximized. We use a number of *f*-divergence measures for capturing the distinction between $P_{A}$ and $Q_{A}$, each with a distinct operational meaning under specific inference problems. For this purpose, we denote the *f*-divergence of $Q_{A}$ from $P_{A}$ by $D_{f}\left(A\right)$, where

(5) $$\begin{array}{c} D_{f}\left(A\right)\;\overset{▵}{=}E_{P_{A}}\left[f\left(\frac{dQ_{A}}{dP_{A}}\right)\right]. \end{array}$$

We use the shorthand $D_{f}\left(A\right)$ for the canonical notation $D_{f}\left(Q_{A}\;∥\;P_{A}\right)$ for emphasizing the dependence on A and for the simplicity in notations. $E_{P_{A}}$ denotes the expectation with respect to $P_{A}$, and f:(0,+∞)→R is a convex function that is strictly convex at 1 and f(1)=0. Strict convexity at 1 ensures that the *f*-divergence between a pair of probability measures is zero if and only if the probability measures are identical. Given the linear dimensionality reduction model in (3), the objective is to solve

(6) $$\begin{array}{cc} P:\;\max_{A\in R^{r\times n}} & \;D_{f}\left(A\right), \end{array}$$

for the following choices of the *f*-divergence measures.

1. *Kullback–Leibler (KL) divergence* for f(t)=tlogt:
  (7) $$\begin{array}{c} D_{\mathrm{KL}}\left(A\right)\;\overset{▵}{=}E_{Q_{A}}\left[\log\frac{dQ_{A}}{dP_{A}}\right]. \end{array}$$
  We also denote the KL divergence from $P_{A}$ to $Q_{A}$ by $D_{\mathrm{KL}}\left(P_{A}\;∥\;Q_{A}\right)$.
2. *Symmetric KL divergence* for f(t)=(t−1)logt:
  (8) $$\begin{array}{c} D_{\mathrm{SKL}}\left(A\right)\;\overset{▵}{=}D_{\mathrm{KL}}\left(Q_{A}\;∥\;P_{A}\right)+D_{\mathrm{KL}}\left(P_{A}\;∥\;Q_{A}\right). \end{array}$$
3. *Squared Hellinger distance* for $f\left(t\right)={\left(1-\sqrt{t}\right)}^{2}$:
  (9) $$\begin{array}{c} H^{2}\left(A\right)\;\overset{▵}{=}\int_{R^{r}}{\left(\sqrt{dQ_{A}}-\sqrt{dP_{A}}\right)}^{2}. \end{array}$$
4. *Total variation distance* for $f\left(t\right)=\frac{1}{2}\cdot \left|t-1\right|$:
  (10) $$\begin{array}{c} d_{\mathrm{TV}}\left(A\right)\;\overset{▵}{=}\frac{1}{2}\int_{R^{r}}\left|dQ_{A}-dP_{A}\right|. \end{array}$$
5. *$\chi^{2}$-divergence* for $f\left(t\right)={\left(t-1\right)}^{2}$:
  (11) $$\begin{array}{c} \chi^{2}\left(A\right)\;\overset{▵}{=}\int_{R^{r}}\frac{{\left(dQ_{A}-dP_{A}\right)}^{2}}{dP_{A}}. \end{array}$$
  We also denote the $\chi^{2}$-divergence from $P_{A}$ to $Q_{A}$ by $\chi^{2}\left(P_{A}\;∥\;Q_{A}\right)$.

## 3. Problem Formulation

In this section, without loss of generality, we focus on the setting where one of the covariance matrices is the identity matrix, and the other one has a covariance matrix Σ in order to avoid complex representations. One key observation is that the design of A under different measures has strong similarities. We first note that, by defining $\bar{A}\;\overset{▵}{=}A\cdot \Sigma_{P}^{1/2}$, $\mu \;\overset{▵}{=}\Sigma_{P}^{-1/2}\cdot \left(\mu_{Q}-\mu_{P}\right)$, and $\Sigma \;\overset{▵}{=}\Sigma_{P}^{-1/2}\cdot \Sigma_{Q}\cdot \Sigma_{P}^{-1/2}$, designing A for maximally distinguishing

(12) $$\begin{array}{c} N\left(A\cdot \mu_{P},A\cdot \Sigma_{P}\cdot A^{⊤}\right)\;\mathrm{and}\;N\left(A\cdot \mu_{Q},A\cdot \Sigma_{Q}\cdot A^{⊤}\right) \end{array}$$

is equivalent to designing $\bar{A}$ for maximally distinguishing

(13) $$\begin{array}{c} N\left(0,\bar{A}\cdot {\bar{A}}^{⊤}\right)\;\mathrm{and}\;N\left(\bar{A}\cdot \mu ,\bar{A}\cdot \Sigma \cdot {\bar{A}}^{⊤}\right). \end{array}$$

Hence, without loss of generality, we focus on the setting where $\mu_{P}=0$, $\Sigma_{P}=I_{n}$, and $\Sigma_{Q}=\Sigma$. Next, we show that determining an optimal design for A can be confined to the class of semi-orthogonal matrices.

**Theorem** **1.** *For every A, there exists a semi-orthogonal matrix $\bar{A}$ such that $D_{f}\left(\bar{A}\right)=D_{f}\left(A\right)$.*

**Proof.**  See Appendix A.    □

This observation indicates that we can reduce the unconstrained problem in (6) to the following constrained problem:

(14) $$\begin{array}{cc} Q:\; & \max_{A\in R^{r\times n}}\;D_{f}\left(A\right)\;s.t.\;A\cdot A^{⊤}=I_{r}. \end{array}$$

We show that the design of A in the case of μ=0, under the considered *f*-divergence measures, directly relates to analyzing the eigenspace of matrix Σ. For this purpose, we denote the non-negative eigenvalues of Σ ordered in the descending order by $\left\{\lambda_{i}:i\in \left[n\right]\right\}$, where for an integer *m* we have defined [m]={1,…,m}. For an arbitrary permutation function π:[n]→[n], we denote the permutation of $\left\{\lambda_{i}:i\in \left[n\right]\right\}$ with respect to π by $\left\{\lambda_{\pi (i)}:i\in \left[n\right]\right\}$. We also denote the eigenvalues of $A\cdot \Sigma \cdot A^{⊤}$ ordered in the descending order by $\left\{\gamma_{i}:i\in \left[r\right]\right\}$. Throughout the analysis, we frequently use Poincaré separation theorem [38] for finding the row space of matrix A with respect to the eigenvalues of Σ.

**Theorem** **2** (Poincaré Separation Theorem)**.** *Let* **Σ** *be a real symmetric n×n matrix and A be a semi-orthogonal r×n matrix. The eigenvalues of* **Σ** *denoted by $\left\{\lambda_{i}:i\in \left[n\right]\right\}$ (sorted in the descending order) and the eigenvalues of $A\cdot \Sigma \cdot A^{⊤}$ denoted by $\left\{\gamma_{i}:i\in \left[r\right]\right\}$ (sorted in the descending order) satisfy*

(15) $$\begin{array}{c} \lambda_{n-(r-i)}\leq \gamma_{i}\leq \lambda_{i},\;\forall i\in \left[r\right]. \end{array}$$

Finally, we define the following functions, which we will refer to frequently throughout the paper:

(16) $$\begin{array}{cc} h_{1}\left(A\right) & \;\overset{▵}{=}A\cdot \Sigma \cdot A^{⊤},\;\;\;\;\;\; \end{array}$$

(17) $$\begin{array}{cc} h_{2}\left(A\right) & \;\overset{▵}{=}\mu^{⊤}\cdot A^{⊤}\cdot A\cdot \mu ,\;\;\;\;\; \end{array}$$

(18) $$\begin{array}{cc} h_{3}\left(A\right) & \;\overset{▵}{=}\mu^{⊤}\cdot A^{⊤}\cdot {\left[h_{1}\left(A\right)\right]}^{-1}\cdot A\cdot \mu . \end{array}$$

In the next sections, we analyze the design of A under different *f*-divergence measures. In particular, in Section 4 and Section 5, we focus on zero-mean Gaussian models for P and Q where we provide an operational interpretation of the measure in the dichotomous mode in (4). Subsequently, we will discuss the generalization to non-zero mean Gaussian models in Section 6.

## 4. Main Results for Zero-Mean Gaussian Models

In this section, we analyze problem Q defined in (14) for each of the *f*-divergence measures separately. Specifically, for each case, we briefly provide an inference problem as a motivating example, in the context of which we relate the optimal performance limit of that inference problem to the *f*-divergence of interest. These analyses are provided in Section 4.1, Section 4.2, Section 4.3, Section 4.4 and Section 4.5. Subsequently, we provide the main results on the optimal design of the linear mapping matrix A in Section 4.6.

### 4.1. Kullback Leibler Divergence

#### 4.1.1. Motivation

The KL divergence, being the expected value of the log-likelihood ratio, captures, at least partially, the performance of a wide range of inference problems. One specific problem whose performance is completely captured by $D_{\mathrm{KL}}\left(A\right)$ is the quickest change-point detection. Consider an observation process (time-series) $\left\{X_{t}:t\in N\right\}$ in which the observations $X_{t}\in R^{n}$ are generated by a distribution with probability measure P specified in (2). This distribution changes to Q at an unknown (random or deterministic) time κ, i.e.,

(19) $$\begin{array}{c} \begin{array}{c} X_{t}∼P\;t<\kappa ,\;\;\mathrm{and}\;\;X_{t}∼Q\;t\geq \kappa \end{array}. \end{array}$$

Change-point detection algorithms sample the observation process sequentially and aim to detect the change point with the minimal delay after it occurs subject to a false alarm constraint. Hence, the two key figures of merit capturing the performance of a sequential change-point detection algorithm are the average detection delay (ADD) and the rate of false alarms. Whether the change-point κ is random or deterministic gives rise to two broad classes of quickest change-point detection problems, namely the Bayesian setting (κ is random) and minimax setting (κ is deterministic). Irrespective of their discrepancies in settings and the nature of performance guarantees, the ADD for the (asymptotically) optimal algorithms are in the form [39]:

(20) $$\begin{array}{c} \mathrm{ADD}∼\frac{c_{1}}{D_{\mathrm{KL}}\left(Q\;∥\;P\right)}. \end{array}$$

Hence, after the linear mapping induced by matrix A, for the ADD, we have

(21) $$\begin{array}{c} \mathrm{ADD}∼\frac{c_{2}}{D_{\mathrm{KL}}\left(Q_{A}\;∥\;P_{A}\right)}, \end{array}$$

where $c_{1}$ and $c_{2}$ are constants specified by the false alarm constraints. Clearly, the design of A that minimizes the ADD will be maximizing the disparity between the pre- and post-change distributions $P_{A}$ and $Q_{A}$, respectively.

#### 4.1.2. Connection between $D_{\mathrm{KL}}$ and A

By noting that A is a semi-orthogonal matrix and recalling that the eigenvalues of $h_{1}\left(A\right)$ are denoted by $\left\{\gamma_{i}:i\in \left[r\right]\right\}$, simple algebraic manipulations simplify $D_{\mathrm{KL}}\left(Q_{A}\;∥\;P_{A}\right)$ to:

(22) $$\begin{array}{cc} D_{\mathrm{KL}}\left(Q_{A}\;∥\;P_{A}\right) & =\frac{1}{2}\left[\log\frac{1}{|h_{1}\left(A\right)|}-r+\mathrm{Tr}\left[h_{1}\left(A\right)\right]+h_{2}\left(A\right)\right]. \end{array}$$

By setting, and leveraging, Theorem 2, the problem of finding an optimal design for A that solves (14) can be found as the solution to:

(23) $$\begin{array}{c} \max_{\left\{\gamma_{i}\;:\;i\in \left[r\right]\right\}}\sum_{i=1}^{r}g_{\mathrm{KL}}\left(\gamma_{i}\right)\;s.t.\;\lambda_{n-(r-i)}\leq \gamma_{i}\leq \lambda_{i}\;\;\forall i\in \left[r\right], \end{array}$$

where we have defined

(24) $$\begin{array}{c} g_{\mathrm{KL}}\left(x\right)\;\overset{▵}{=}\frac{1}{2}\left(x-\log x-1\right). \end{array}$$

Likewise, finding the optimal design for A that optimizes $D_{\mathrm{KL}}\left(P_{A}\;∥\;Q_{A}\right)$ when μ=0 can be found by replacing $g_{\mathrm{KL}}\left(\gamma_{i}\right)$ by $g_{\mathrm{KL}}\left(\frac{1}{\gamma_{i}}\right)$ in (23). In either case, the optimal design of A is constructed by choosing *r* eigenvectors of Σ as the rows of A. The results and observations are formalized in Section 4.6.

### 4.2. Symmetric KL Divergence

#### 4.2.1. Motivation

The KL divergence discussed in Section 4.1 is an asymmetric measure of separation between two probability measures. It is symmetrized by adding two directed divergence measures in reverse directions. The symmetric KL divergence has applications in model selection problems in which the model selection criteria is based on a measure of disparity between the true model and the approximating models. As shown in Reference [40], using the symmetric KL divergence outperforms the individual directed KL divergences since it better reflects the risks associated with underfitting and overfitting of the models, respectively.

#### 4.2.2. Connection between $D_{\mathrm{SKL}}$ and A

For a given A, the symmetric KL divergence of interest specified in (8) is given by

(25) $$\begin{array}{c} D_{\mathrm{SKL}}\left(A\right)=\frac{1}{2}\cdot \left[\mathrm{Tr}\left({\left[h_{1}\left(A\right)\right]}^{-1}+h_{1}\left(A\right)\right)+h_{2}\left(A\right)+h_{3}\left(A\right)\right]-r. \end{array}$$

By setting μ=0, and leveraging Theorem 2, the problem of finding an optimal design for A that solves (14) can be found as the solution to:

(26) $$\begin{array}{c} \max_{\left\{\gamma_{i}\;:\;i\in \left[r\right]\right\}}\sum_{i=1}^{r}g_{\mathrm{SKL}}\left(\gamma_{i}\right)\;s.t.\;\lambda_{n-(r-i)}\leq \gamma_{i}\leq \lambda_{i}\;\;\forall i\in \left[r\right], \end{array}$$

where we have defined

(27) $$\begin{array}{c} g_{\mathrm{SKL}}\left(x\right)\;\overset{▵}{=}\frac{1}{2}\left(x+\frac{1}{x}-2\right). \end{array}$$

### 4.3. Squared Hellinger Distance

#### 4.3.1. Motivation

Squared Hellinger distance facilitates analysis in high dimensions, especially when other measures fail to take closed-form expressions. We will discuss an important instance of this in the next subsection in the analysis of $d_{\mathrm{TV}}$. Squared Hellinger distance is symmetric, and it is confined in the range [0,2].

#### 4.3.2. Connection between $H^{2}$ and A

For a given matrix A, we have the following closed-form expression:

(28) $$\begin{array}{c} H^{2}\left(A\right)=\;\begin{array}{cc} & 2-2\frac{|4\cdot h_{1}{\left(A\right)|}^{\frac{1}{4}}}{|h_{1}\left(A\right)+I_{r}{|}^{\frac{1}{2}}}\cdot \exp\left(-\frac{\mu^{⊤}\cdot A^{⊤}\cdot {\left[h_{1}\left(A\right)+I_{r}\right]}^{-1}\cdot A\cdot \mu }{4}\right). \end{array} \end{array}$$

By setting μ=0, and leveraging Theorem 2, the problem of finding an optimal design for A that solves (14) can be found as the solution to:

(29) $$\begin{array}{c} \max_{\left\{\gamma_{i}\;:\;i\in \left[r\right]\right\}}\prod_{i=1}^{r}g_{H}\left(\gamma_{i}\right)\;s.t.\;\lambda_{n-(r-i)}\leq \gamma_{i}\leq \lambda_{i}\;\;\forall i\in \left[r\right], \end{array}$$

where we have defined

(30) $$\begin{array}{c} g_{H}\left(x\right)\;\overset{▵}{=}\frac{{\left(x+1\right)}^{2}}{x}. \end{array}$$

### 4.4. Total Variation Distance

#### 4.4.1. Motivation

The total variation distance appears as the key performance metric in binary hypothesis testing and in high-dimensional inference, e.g., Le Cam’s method for the binary quantization and testing of the individual dimensions (which is in essence binary hypothesis testing). In particular, for the simple binary hypothesis testing model in (65), the minimum total probability of error (sum of type-I and type-II error probabilities) is related to the total variation $d_{\mathrm{TV}}\left(A\right)$. Specifically, for a decision rule $d:X\to \{H_{0},H_{1}\}$, the following holds:

(31) $$\begin{array}{c} \underset{d}{\inf}\;\left[P_{A}\left(d=H_{1}\right)+Q_{A}\left(d=H_{0}\right)\right]=1-d_{\mathrm{TV}}\left(A\right). \end{array}$$

The total variation between two Gaussian distributions does not have a closed-form expression. Hence, unlike the other settings, an optimal solution to (6) in this context cannot be obtained analytically. Alternatively, in order to gain intuition into the structure of a near optimal matrix A, we design A such that it optimizes known bounds on $d_{\mathrm{TV}}\left(A\right)$. In particular, we use two sets of bounds on $d_{\mathrm{TV}}\left(A\right)$. One set is due to bounding it via the Hellinger distance, and another set is due to a recent study that established upper and lower bounds that are identical up to a constant factor [41].

#### 4.4.2. Connection between $d_{\mathrm{TV}}$ and A

*(1) Bounding by Hellinger Distance:* The total variation distance can be bounded by the Hellinger distance according to

(32) $$\begin{array}{c} \frac{1}{2}H^{2}\left(A\right)\leq d_{\mathrm{TV}}\left(A\right)\leq H\left(A\right)\sqrt{1-\frac{H^{2}\left(A\right)}{4}}. \end{array}$$

It can be readily verified that these bounds are monotonically increasing with $H^{2}\left(A\right)$ in the interval [0,2]. Hence, they are maximized simultaneously by maximizing the squared Hellinger distance as discussed in Section 4.3. We refer to this bound as the Hellinger bound.

*(2) Matching Bounds up to a Constant:* The second set of bounds that we used are provided in Reference [41]. These bounds relate the total variation between two Gaussian models to the Frobenius norm (FB) of a matrix related to their covariance matrices. Specifically, these FB-based bounds on the total variation $d_{\mathrm{TV}}\left(A\right)$ are given by

(33) $$\begin{array}{c} \frac{1}{100}\leq \frac{d_{\mathrm{TV}}\left(A\right)}{\min\{1,\sqrt{\sum_{i=1}^{r}g_{\mathrm{TV}}\left(\gamma_{i}\right)}\}}\leq \frac{3}{2}, \end{array}$$

where we have defined

(34) $$\begin{array}{c} g_{\mathrm{TV}}\left(x\right)\;\overset{▵}{=}{\left(\frac{1}{x}-1\right)}^{2}. \end{array}$$

Since the lower and upper bounds on $d_{\mathrm{TV}}\left(A\right)$ are identical up to a constant, they will be maximized by the same design of A.

### 4.5. $\chi^{2}$-Divergence

#### 4.5.1. Motivation

$\chi^{2}$-divergence appears in a wide range of statistical estimation problems for the purpose of finding a lower bound on the estimation noise variance. For instance, consider the canonical problem of estimating a latent variable θ from the observed data *X*, and denote two candidate estimates by p(X) and q(X). Define P and Q as the probability measures of p(X) and q(X), respectively. According to the Hammersly-Chapman-Robbins (HCR) bound on the quadratic loss function, for any estimator $\hat{\theta }$, we have

(35) $$\begin{array}{c} {\mathrm{var}}_{\theta }\left(\hat{\theta }\right)\geq \underset{p\neq q}{\sup}\frac{{\left[E_{Q}\left[q\left(X\right)\right]-E_{P}\left[p\left(X\right)\right]\right]}^{2}}{\chi^{2}\left(Q\;∥\;P\right)}, \end{array}$$

which, for unbiased estimators *p* and *q*, simplifies to the Cramér-Rao lower bound

(36) $$\begin{array}{c} {\mathrm{var}}_{\theta }\left(\hat{\theta }\right)\geq \underset{p\neq q}{\sup}\frac{{\left(q-p\right)}^{2}}{\chi^{2}\left(Q\;∥\;P\right)}, \end{array}$$

depending on P and Q through their $\chi^{2}$-divergence. Besides the applications to estimation problems, $\chi^{2}$ is easier to compute compared to some of other *f*-divergence measures (e.g., total variation). Specifically, for product distributions $\chi^{2}$ tensorizes to be expressed in terms of the one-dimensional components that are easier to compute than the KL divergence and TV variation distance. Hence, a combination of bounding other measures with $\chi^{2}$ and then analyzing $\chi^{2}$ appears in a wide range of inference problems.

#### 4.5.2. Connection between $\chi^{2}$ and A

By setting μ=0, for a given matrix A, from (11), we have the following closed-form expression:

(37) $$\begin{array}{cc} \chi^{2}\left(A\right) & =\frac{1}{|h_{1}\left(A\right)|\sqrt{|2{\left(h_{1}\left(A\right)\right)}^{-1}-I_{r}|}}-1 \end{array}$$

(38) $$\begin{array}{cc} & =\prod_{i=1}^{r}g_{\chi_{1}}\left(\gamma_{i}\right)-1,\;\;\;\;\; \end{array}$$

where we have defined

(39) $$\begin{array}{c} g_{\chi_{1}}\left(x\right)\;\overset{▵}{=}\frac{1}{\sqrt{x(2-x)}}. \end{array}$$

As we show in Appendix C, for $\chi^{2}\left(A\right)$ to exist (i.e., be finite), all the eigenvalues $\left\{\lambda_{i}:i\in \left[r\right]\right\}$ should fall in the interval (0,2). Subsequently, finding the optimal design for A that optimizes $\chi^{2}\left(P_{A}\;∥\;Q_{A}\right)$ when μ=0 can be done by replacing $g_{\chi_{1}}$ in (38) by $g_{\chi_{2}}$, which is given by

(40) $$\begin{array}{c} g_{\chi_{2}}\left(x\right)\;\overset{▵}{=}\sqrt{\frac{x^{2}}{2x-1}}. \end{array}$$

Based on this, and by following a similar line of argument as in the case of the KL divergence, designing an optimal A reduces to identifying a subset of the eigenvalues of Σ and assigning their associated eigenvectors as the rows of matrix A. These observations are formalized in Section 4.6.

### 4.6. Main Results

In this section, we provide analytical closed-form solutions to design optimal matrices A for the following *f*-divergence measures: $D_{\mathrm{KL}}$, $D_{\mathrm{SKL}}$, $H^{2}$, and $\chi^{2}$. The total variation measure $d_{\mathrm{TV}}$ does not admit a closed-form for Gaussian models. In this case, we provide a design for A that optimizes the bound we have provided for $d_{\mathrm{TV}}$ in Section 4.4. Due to their structural similarities of the results, we group and treat $D_{\mathrm{KL}}$, $D_{\mathrm{SKL}}$, and $d_{\mathrm{TV}}$ in Theorem 3. Similarly, we group and treat $H^{2}$ and $\chi^{2}$ in Theorem 4.

**Theorem** **3** ($D_{\mathrm{KL}}$, $D_{\mathrm{SKL}}$, $d_{\mathrm{TV}}$)**.** *For a given function g:R→R, define the permutations:*

(41) $$\begin{array}{c} \pi^{*}\;\overset{▵}{=}\arg\max_{\pi }\sum_{i=1}^{r}g\left(\lambda_{\pi (i)}\right). \end{array}$$

*Then, for $D_{f}\left(A\right)\in \left\{D_{\mathrm{KL}}\left(A\right),D_{\mathrm{SKL}}\left(A\right),d_{\mathrm{TV}}\left(A\right)\right\}$ and functions $g_{f}$$\in \{g_{\mathrm{KL}},g_{\mathrm{SKL}},g_{\mathrm{TV}}\}$:*

1. *For maximizing $D_{f}$, set $g=g_{f}$ and select the eigenvalues of $A\Sigma A^{⊤}$ as*
  (42) $$\begin{array}{c} \gamma_{i}=\lambda_{\pi^{*}\left(i\right)},\;for\;i\in \left[r\right]. \end{array}$$
2. *Row i∈[r] of matrix A is the eigenvector of***Σ***associated with the eigenvalue $\gamma_{i}$.*

**Proof.**  See Appendix B.    □

By further leveraging the structures of functions $g_{\mathrm{KL}},g_{\mathrm{SKL}}$, and $g_{\mathrm{TV}}$, we can simplify approaches for designing the matrix A. Specifically, note that the functions $g_{\mathrm{KL}},g_{\mathrm{SKL}},and\;g_{\mathrm{TV}}$ are all strictly convex functions taking their global minima at x=1. Based on this, we have the following observations.

**Corollary** **1** ($D_{\mathrm{KL}}$, $D_{\mathrm{SKL}}$, $d_{\mathrm{TV}}$)**.** *For maximizing $D_{f}\left(A\right)\in \left\{D_{\mathrm{KL}}\left(A\right),D_{\mathrm{SKL}}\left(A\right),d_{\mathrm{TV}}\left(A\right)\right\}$, when $\lambda_{n}\geq 1$, we have $\gamma_{i}=\lambda_{i}$ for all i∈[r], and the rows of A are eigenvectors of* **Σ** *associated with its r largest eigenvalues, i.e., $\left\{\lambda_{i}:i\in \left[r\right]\right\}$.*

**Corollary** **2** ($D_{\mathrm{KL}}$, $D_{\mathrm{SKL}}$, $d_{\mathrm{TV}}$)**.** *For maximizing $D_{f}\left(A\right)\in \left\{D_{\mathrm{KL}}\left(A\right),D_{\mathrm{SKL}}\left(A\right),d_{\mathrm{TV}}\left(A\right)\right\}$, when $\lambda_{1}\leq 1$, we have $\gamma_{i}=\lambda_{n-r+i}$ for all i∈[r], and the rows of A are eigenvectors of* **Σ** *associated with its r smallest eigenvalues, i.e., $\left\{\lambda_{i}:i\in \left\{n-r+1,\cdots ,n\right\}\right\}$.*

**Remark** **1.** *In order to maximize $D_{f}\left(A\right)\in \left\{D_{\mathrm{KL}}\left(A\right),D_{\mathrm{SKL}}\left(A\right),d_{\mathrm{TV}}\left(A\right)\right\}$ when $\lambda_{n}\leq 1\leq \lambda_{1}$, finding the best permutation of eigenvalues involves sorting all the n eigenvalues $\lambda_{i}$’s and subsequently performing r comparisons as illustrated in Algorithm 1. This amounts to O(n·log(n)) time complexity instead of O(n·log(r)) time complexity involved in determining the design for A in the case of Corollaries 1 and 2, which require finding the r extreme eigenvalues in determining the design for $\pi^{*}$.*

**Remark** **2.** *The optimal design of A often does not involve being aligned with the largest eigenvalues of the covariance matrix* **Σ** *, which is in contrast to some of the key approaches to linear dimensionality reduction that generally perform linear mapping along the eigenvectors associated with the largest eigenvalues of the covariance matrix. When the eigenvalues of* **Σ** *are all smaller than 1, in particular, A will be designed by choosing eigenvectors associated with the smallest eigenvalues of* **Σ** *in order to preserve largest separability.*

Next, we provide the counterpart results for the $H^{2}$ and $\chi^{2}$-divergence measures. Their major distinction from the previous three measures is that, for these two, $D_{f}\left(A\right)$ can be decomposed into a product of individual functions of the eigenvalues $\left\{\gamma_{i}:i\in \left[r\right]\right\}$. Next, we provide the counterparts of Theorem 3 and Corollaries 1 and 2 for $H^{2}$ and $\chi^{2}$.

**Theorem** **4** ($H^{2}$, $\chi^{2}$). *For a given function g:R→R, define the permutations:*

(43) $$\begin{array}{c} \pi^{*}\;\overset{▵}{=}\arg\max_{\pi }\prod_{i=1}^{r}g\left(\lambda_{\pi (i)}\right). \end{array}$$

*Then, for $D_{f}\left(A\right)\in \left\{H^{2}\left(A\right),\chi^{2}\left(A\right),\chi^{2}\left(P_{A}\;∥\;Q_{A}\right)\right\}$ and functions $g_{f}\in \left\{g_{H},g_{\chi_{1}},g_{\chi_{2}}\right\}$:*

1. *For maximizing $D_{f}$, set $g=g_{f}$ and select the eigenvalues of $A\Sigma A^{⊤}$ as*
  (44) $$\begin{array}{c} \gamma_{i}=\lambda_{\pi^{*}\left(i\right)},\;for\;i\in \left[r\right]. \end{array}$$
2. *Row i∈[r] of matrix A is the eigenvector of***Σ***associated with the eigenvalue $\gamma_{i}$.*

**Proof.**  See Appendix C.    □

Next, note that $g_{H}$ is a strictly convex function taking its global minimum at x=1. Furthermore, $g_{\chi_{i}}$ for i∈[2] are strictly convex over (0,2) and take their global minimum at x=1.

**Corollary** **3** ($H^{2}$, $\chi^{2}$)**.** *For maximizing $D_{f}\left(A\right)\in \left\{H^{2}\left(A\right),\chi^{2}\left(A\right),\chi^{2}\left(P_{A}\;∥\;Q_{A}\right)\right\}$, when $\lambda_{n}\geq 1$, we have $\gamma_{i}=\lambda_{i}$ for all i∈[r], and the rows of A are eigenvectors of* **Σ** *associated with its r largest eigenvalues, i.e., $\left\{\lambda_{i}:i\in \left[r\right]\right\}$.*

**Corollary** **4** ($H^{2}$, $\chi^{2}$)**.** *For maximizing $D_{f}\left(A\right)\in \left\{H^{2}\left(A\right),\chi^{2}\left(A\right),\chi^{2}\left(P_{A}\;∥\;Q_{A}\right)\right\}$, when $\lambda_{1}\leq 1$, we have $\gamma_{i}=\lambda_{n-r+i}$ for all i∈[r], and the rows of A are eigenvectors of* **Σ** *associated with its r smallest eigenvalues, i.e., $\left\{\lambda_{i}:i\in \left\{n-r+1,\cdots ,n\right\}\right\}$.*

**Algorithm 1:** Optimal Permutation $\pi^{*}$ When $\lambda_{n}\leq 1\leq \lambda_{1}$
1:Initialize i←n, j←1, $p_{k}\leftarrow \lambda_{k}\;\forall k\in \left\{i,j\right\}$, $\pi^{*}\leftarrow \emptyset$2:Sort the eigenvalues of Σ in descending order $\left\{\lambda_{k}:k\in \left[n\right]\right\}$3:
**while** $|\pi^{*}|\neq r$ **do**
4:  **if** $g_{f}\left(p_{i}\right)>g_{f}\left(p_{j}\right)$ **then**5:    $\pi^{*}\leftarrow \pi^{*}\cup \left\{p_{i}\right\}$6:    i←i−17:  **else**8:    $\pi^{*}\leftarrow \pi^{*}\cup \left\{p_{j}\right\}$9:    j←j+110:  **end if**11:
**end while**
12:
**return** $\pi^{*}$




Finally, we remark that, unlike the other measures, total variation does not admit a closed-form, and we used two sets of tractable bounds to analyze this case of total variations. By comparing the design of A based on different bounds, we have the following observation.

**Remark** **3.** *We note that both sets of bounds lead to the same design of A when either $\lambda_{1}\leq 1$ or $\lambda_{n}\geq 1$. Otherwise, each will be selecting a different set of the eigenvectors of* **Σ** *to construct A according to the functions*

(45) $$\begin{array}{c} g_{H}\left(x\right)=\frac{{\left(x+1\right)}^{2}}{x}\;\mathrm{versus}\;g_{\mathrm{TV}}\left(x\right)={\left(\frac{1}{x}-1\right)}^{2}. \end{array}$$

## 5. Zero-Mean Gaussian Models–Simulations

### 5.1. KL Divergence

In this section, we show gains of the above analysis for the KL divergence measure $D_{\mathrm{KL}}\left(A\right)$ through simulations on a change-point detection problem. We focus on the minimax setting in which the change-point κ is deterministic. The objective is to detect a change in the stochastic process $X_{t}$ with minimal delay after the change in the probability measure occurs at κ and define τ∈N as the time that we can form a confident decision. A canonical model to quantify the decision delay is the conditional average detection delay (CADD) due to Pollak [42]

(46) $$\begin{array}{cc} \mathrm{CADD}(\tau ) & \;\overset{▵}{=}\underset{\kappa \geq 1}{\sup}\;E_{\kappa }\;\left[\tau -\kappa \;|\;\tau \geq \kappa \right], \end{array}$$

where $E_{\kappa }$ is the expectation with respect to the probability distribution when the change happens at time κ. The objective of this formulation is to optimize the decision delay for the worst-case realization of the random change-point κ (that is, the change-point realization that leads to the maximum decision delay), while the constraints on the false alarm rate are satisfied. In this formulation, this worst-case realization is κ=1, in which case all the data points are generated from the post-change distribution. In the minimax setting, a reasonable measure of false alarms is the mean-time to false alarm, or its reciprocal, which is the false alarm rate (FAR) defined as

(47) $$\begin{array}{cc} \mathrm{FAR}(\tau ) & \;\overset{▵}{=}\frac{1}{E_{\infty }\left[\tau \right]}\;, \end{array}$$

where $E_{\infty }$ is the expectation with respect to the distribution when a change never occurs, i.e., $\kappa \;\overset{▵}{=}\infty$. A standard approach to balance the trade-off between decision delay and false alarm rates involves solving [42]

(48) $$\begin{array}{c} \min_{\tau }\mathrm{CADD}\left(\tau \right)\;s.t.\;\mathrm{FAR}\left(\tau \right)\leq \alpha , \end{array}$$

where $\alpha \in R_{+}$ controls the rate of false alarms. For the quickest change-point detection formulation in (48), the popular cumulative sum (CuSum) test generates the optimal solutions, involving computing the following test statistic:

(49) $$\begin{array}{c} W\left[t\right]\;\overset{▵}{=}\max_{1\leq k\leq t+1}\sum_{i=k}^{t}\log\left(\frac{dQ_{A}\left(X_{i}\right)}{dP_{A}\left(X_{i}\right)}\right). \end{array}$$

Computing W[t] follows a convenient recursion given by

(50) $$\begin{array}{c} W\left[t\right]\;\overset{▵}{=}{\left(W\left[t-1\right]+\log\left(\frac{dQ_{A}\left(X_{t}\right)}{dP_{A}\left(X_{t}\right)}\right)\right)}^{+}, \end{array}$$

where W[0]=0. The CuSum statistic declares a change at a stopping time τ given by

(51) $$\begin{array}{c} \tau \;\overset{▵}{=}\inf\left\{t\geq 1:W\left[t\right]>C\right\}, \end{array}$$

where *C* is chosen such that the constraint on FAR(τ) in (48) is satisfied.

In this setting, we consider two zero-mean Gaussian models with the following pre- and post-linear dimensionality reduction structures:

(52) $$\begin{array}{c} \begin{array}{cc} P:\;\; & N\left(0,I_{n}\right)\;\mathrm{and}\;Q:\;\;N\left(0,\Sigma \right)\\ P_{A}:\;\; & N\left(0,I_{r}\right)\;\mathrm{and}\;Q_{A}:\;\;N\left(0,h_{1}\left(A\right)\right) \end{array}, \end{array}$$

where the covariance matrix Σ is generated randomly, and its eigenvalues are sampled from a uniform distribution. In particular, for the original data dimension *n*, ∗⌈0.9n⌉ eigenvalues are sampled such that $\left\{\lambda_{i}∼U\left(0.064,1\right)\right\}$, and the remaining eigenvalues are sampled such that $\left\{\lambda_{i}∼U\left(1,4.24\right)\right\}$. We note that this is done since the objective function lies in the same range for the eigenvalues within the range [0.0649,1] and [1,4.24]. In order to consider the worst case detection delay, we set κ=1 and generate stochastic observations according to the model described in (52) that follows the change-point detection model in (19). For every random realization of covariance matrix Σ, we run the CuSum statistic (50), where we generate A according to the following two schemes:

*(1) Largest eigen modes:* In this scheme, the linear map A is designed such that its rows are eigenvectors associated with the *r* largest eigenvalues of Σ.

*(2) Optimal design:* In this scheme, the linear map A is designed such that its rows are eigenvectors associated with *r* eigenvalues of Σ that maximize $D_{\mathrm{KL}}\left(A\right)$ according to Theorem 3.

**Figure 1:**
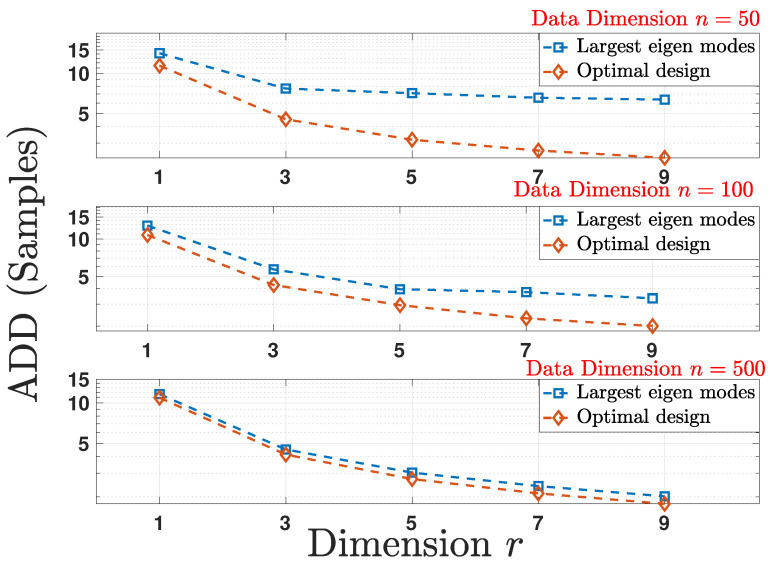
Comparison of the average detection delay (ADD) under the optimal design and largest eigen modes schemes for multiple reduced data dimensions *r* as a function of original data dimension *n* for a fixed false alarm rate (FAR) which is equal to 1/5000.

In order to evaluate and compare the performance of the two schemes, we compute the ADD obtained by running a Monte-Carlo simulation over 5000 random realizations of the stochastic process $X_{t}$ following the change-point detection model in (19) for every random realization of Σ and for each reduced dimension 1≤r≤9. The detection delays obtained are then averaged again over 100 random realizations of covariance matrices Σ for each reduced dimension *r*. Figure 1 shows the plot for ADD versus *r* for multiple initial data dimension *n* and for a fixed $\mathrm{FAR}=\frac{1}{5000}$. Owing to the dependence on $D_{\mathrm{KL}}\left(A\right)$ given in (21), the delay associated with the optimal linear mapping in Theorem 3 achieves better performance.

### 5.2. Symmetric KL Divergence

**Figure 2:**
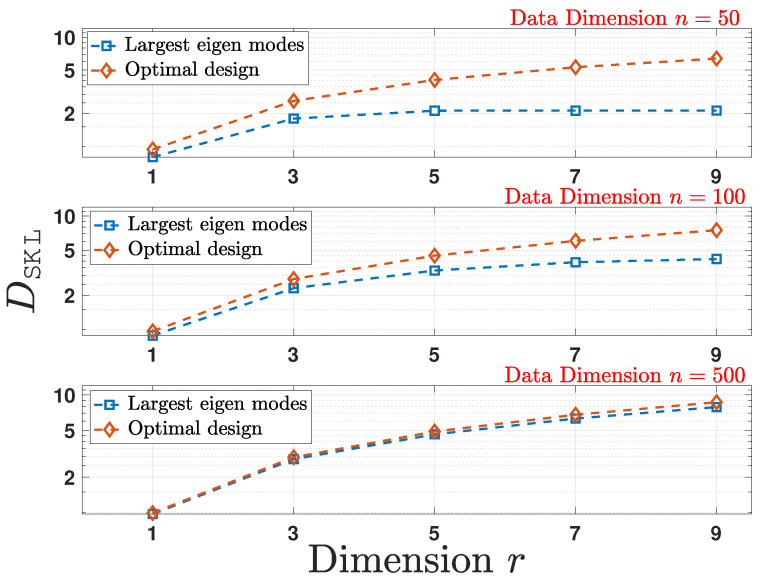
Comparison of the empirical average computed for the optimal design and largest eigen modes schemes for multiple reduced data dimensions *r* as a function of original data dimension *n*.

In this section, we show the gains of the analysis by numerically computing $D_{\mathrm{SKL}}\left(A\right)$. We follow the pre- and post-linear dimensionality reduction structures given in (52), where the covariance matrix Σ is randomly generated following the setup used in Section 5.1. As plotted in Figure 2, by choosing the design scheme for $D_{\mathrm{SKL}}\left(A\right)$ according to Theorem 3, the optimal design outperforms other schemes.

### 5.3. Squared Hellinger Distance

We consider a Bayesian hypothesis testing problem given class a priori parameters $p_{P_{A}}$, $p_{Q_{A}}$ and Gaussian class conditional densities for the linear dimensionality reduction model in (52). Without loss of generality, we assume a 0–1 loss function associated with misclassification for the hypothesis test. In order to quantify the performance of the Bayes decision rule, it is imperative to compute the associated probability of error, also known as the Bayes error, which we denote by $P_{e}$. Since, in general, computing $P_{e}$ for the optimal decision rule for multivariate Gaussian conditional densities is intractable, numerous techniques have been devised to bound $P_{e}$. Owing to its simplicity, one of the most commonly employed metric is the Bhattacharyya coefficient given by

(53) $$\begin{array}{c} \mathrm{BC}\left(A\right)\;\overset{▵}{=}\int_{R^{r}}\sqrt{dP_{A}\cdot dQ_{A}}. \end{array}$$

The metric in (53) facilitates upper bounding the error probability as

(54) $$\begin{array}{c} P_{e}\leq \sqrt{p_{P_{A}}\;p_{Q_{A}}}\cdot \mathrm{BC}\left(A\right), \end{array}$$

which is widely referred to as the Bhattacharrya bound. Relevant to this study is that the squared Hellinger distance is related to the Bhattacharyya coefficient in (53) through

(55) $$\begin{array}{c} H^{2}\left(A\right)=2-\mathrm{BC}\left(A\right). \end{array}$$

**Figure 3:**
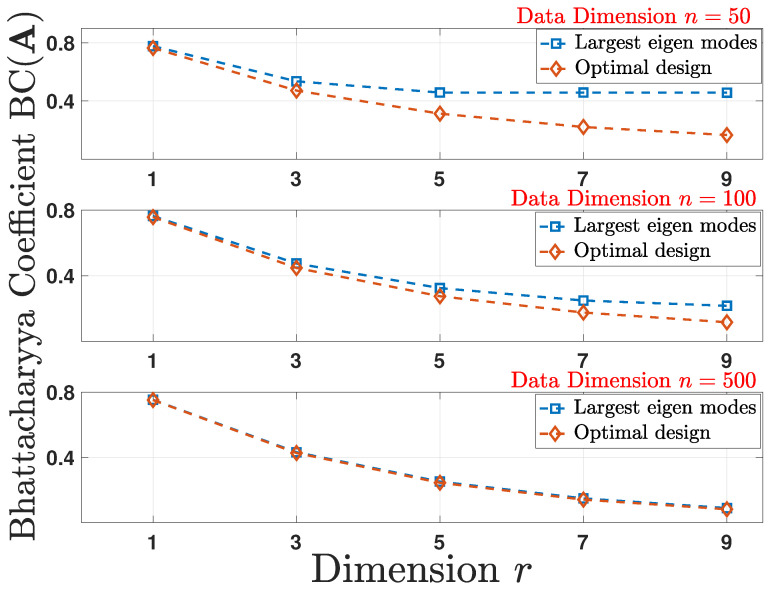
Comparison of the empirical average of the Bhattacharyya coefficient BC(A) under optimal design and largest eigen modes schemes for multiple reduced data dimensions *r* as a function of original data dimension *n*.

Hence, maximizing the Hellinger distance $H^{2}\left(A\right)$ results in a tighter bound on $P_{e}$ from (54). To show the performance numerically, we compute the BC(A) via (55). For the pre- and post-linear dimensionality reduction structures as given in (52), the covariance matrix Σ is randomly generated following the setup used in Section 5.1. As plotted in Figure 3, by employing the design scheme according to Theorem 4, the optimal design results in a smaller BC(A) and, hence, a tighter upper bound on $P_{e}$ in comparison to other schemes.

### 5.4. Total Variation Distance

Consider a binary hypothesis test with Gaussian class conditional densities following the model in (52) and equal class a priori probabilities, i.e., $p_{P_{A}}=p_{Q_{A}}$. We define $c_{ij}$ as the cost associated with deciding in favor of $H_{i}$ when the true hypothesis is $H_{j}$ such that 0≤i,j≤1, and denote the densities associated with measures $P_{A}$, $Q_{A}$ by $f_{P_{A}}$ and $f_{Q_{A}}$, respectively. Without loss of generality, we assume a 0–1 loss function such that $c_{ij}=1\;\forall \;i\neq j$ and $c_{ii}=0\;\forall \;i$. The optimal Bayes decision rule that minimizes the error probability is given by

(56) $$\begin{array}{c} \frac{f_{P_{A}}\left(x\right)}{f_{Q_{A}}\left(x\right)}\;\;\overset{d=H_{1}}{\underset{d=H_{0}}{≶}}\;\;1. \end{array}$$

**Figure 4:**
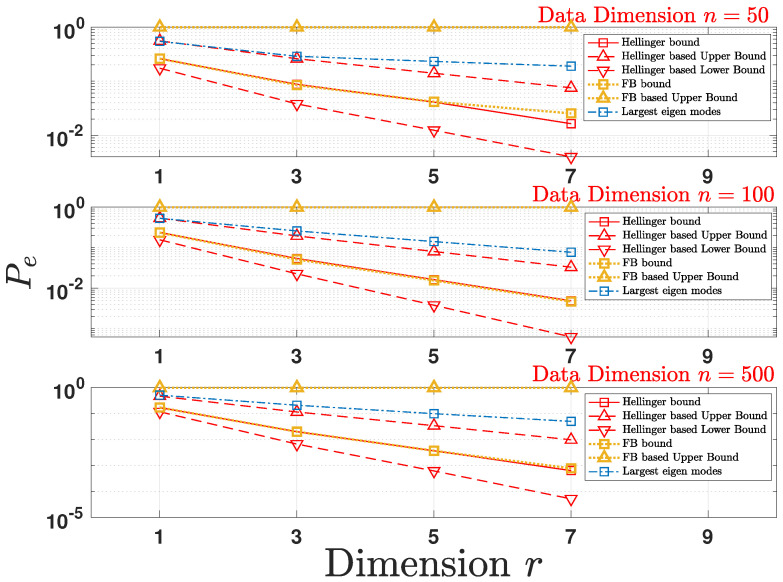
Comparing the logarithm of the empirical average value for $P_{e}$ under the two bounds on $d_{\mathrm{TV}}\left(A\right)$ (Hellinger-based and Frobenius norm (FB)-based) with the largest eigen modes scheme for multiple projected data dimensions *r* as a function of initial data dimension *n*.

Since the total variation distance cannot be computed in closed-form, we numerically compute the error probability $P_{e}$ under the two bounds (Hellinger-based and FB-based) introduced in Section 4.4.2 to quantify the performance of the design of matrix A for the underlying inference problem. The covariance matrix Σ is randomly generated following the setup used in Section 5.1. As plotted in Figure 4, by optimizing the Hellinger-based bound according to Theorem 4 and optimizing the FB-based bound according to Theorem 3, the two design schemes achieve a smaller $P_{e}$. We further observe that the bounds due to FB-based are loose in comparison to Hellinger-based bounds. Therefore, we choose not to plot the lower bound on $P_{e}$ for the FB-based bounds in Figure 4.

### 5.5. $\chi^{2}$-Divergence

**Figure 5:**
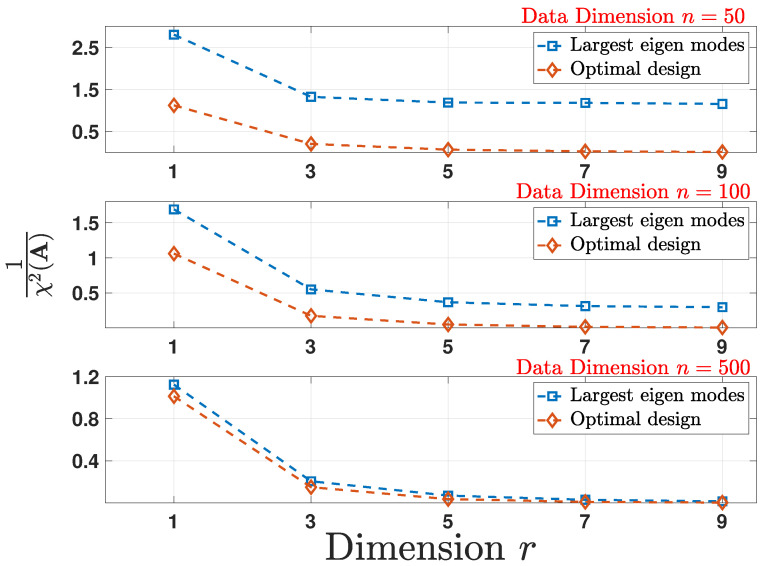
Comparison of the lower bound on noise variance given by $\frac{1}{\chi^{2}\left(A\right)}$ under the optimal and largest eigen modes schemes for multiple reduced data dimensions *r* as a function of original data dimension *n*.

In this section, we show the gains of the proposed analysis through numerical evaluations by numerically computing $\chi^{2}\left(A\right)$, to find a lower bound on the noise variance ${\mathrm{var}}_{\theta }\left(\hat{\theta }\right)$ up to a constant. Following the pre- and post-linear dimensionality reduction structures given in (52), the covariance matrix Σ is randomly generated following the setup used in Section 5.1. As shown in Figure 5, constructing the optimal design according to Theorem 4 achieves a tighter lower bound in comparison to the other scheme.

## 6. General Gaussian Models

In the previous section, we focused on μ=0. When μ≠0, optimizing each *f*-divergence measure under the semi-orthogonality constraint does not render closed-form expressions. Nevertheless, to provide some intuitions, we provide a numerical approach to the optimal design of A, which might also enjoy some *local* optimality guarantees. To start, note that the feasible set of solutions given by $M_{n}^{r}\;\overset{▵}{=}\left\{A\in R^{r\times n}:A\cdot A^{⊤}=I_{r}\right\}$ owing to the orthogonality constraints in Q is often referred to as the Stiefel manifold. Therefore, solving Q requires designing algorithms that optimize the objective while preserving manifold constraints during iterations.

We employ the method of Lagrange multipliers to formulate the Lagrangian function. By denoting the matrix of Lagrangian multipliers by $L\in R^{r\times r}$, the Lagrangian function of problem (14) is given by

(57) $$\begin{array}{c} L\left(A,L\right)=D_{f}\left(A\right)+\langle L,A\cdot A^{⊤}-I_{r}\rangle . \end{array}$$

From the first order optimality condition, for any local maximizer $A^{*}$ of (14), there exists a Lagrange multiplier $L^{*}$ such that

(58) $$\begin{array}{c} \nabla_{A}L\left(A,L\right){|}_{A^{*},L^{*}}=0, \end{array}$$

where we denote the partial derivative with respect to A by $\nabla_{A}$. In what follows, we iterate the design mapping A using the gradient ascent algorithm in order to find a solution for A. As discussed in the next subsection, this solution is guaranteed to be at least locally optimal.

### 6.1. Optimizing via Gradient Ascent

We use an iterative gradient ascent-based algorithm to find the local maximizer of $D_{f}\left(A\right)$ such that $A\in M_{n}^{r}$. The gradient ascent update at any given iteration k∈N is given by

(59) $$\begin{array}{c} A^{k+1}=A^{k}+\alpha \cdot \nabla_{A}L\left(A,L\right){|}_{A^{k}}. \end{array}$$

Note that, following this update, since the new point $A^{k+1}$ in (59) may not satisfy the semi-orthogonality, i.e., $A^{k+1}\notin M_{n}^{r}$, it is imperative to establish a relation between the multipliers L and $A^{k}$ in every iteration *k* to ensure a constraint-preserving update scheme. In particular, to enforce the semi-orthogonality constraint on $A^{k+1}$, a relationship between the multipliers and the gradients in every iteration *k* is derived. Following a similar line of analysis for gradient descent in Reference [43], the relationship between multipliers and the gradients is provided in Appendix E. More details on the analysis of the update scheme can be found in Reference [43], and a detailed discussion on the convergence guarantees of classical steepest descent update schemes adapted to semi-orthogonality constraints can be found in Reference [44].

In order to simplify $\nabla_{A}L\left(A,L\right)$ and state the relationships, we define $\Lambda \;\overset{▵}{=}L+L^{⊤}$ and subsequently find a relationship between Λ and $A^{k}$ in every iteration *k*. This is obtained by right-multiplying (59) by $A^{k+1}$ and solving for Λ that enforces the semi-orthogonality constraint on $A^{k+1}$. To simplify the analysis, we take a finite Taylor series expansion of Λ around α=0 and choose α such that the error in forcing the constraint is a good approximation of the gradient of the objective subjected to $A\cdot A^{⊤}=I_{r}$. As derived in the Appendix E, by simple algebraic manipulations, it can be shown that the matrices $\Lambda_{0},\Lambda_{1}$, and $\Lambda_{2}$, for which the finite Taylor series expansion of $\Lambda \approx \Lambda_{0}+\alpha \cdot \Lambda_{1}+\alpha^{2}\cdot \Lambda_{2}$ is a good approximation of the constraint, are given by

(60) $$\begin{array}{cc} \Lambda_{0} & \;\overset{▵}{=}-\frac{1}{2}\left[\nabla_{A}D_{f}\left(A\right)\cdot {\left(A\right)}^{⊤}+A\cdot {\nabla_{A}D_{f}\left(A\right)}^{⊤}\right],\;\;\;\;\;\;\;\;\;\;\; \end{array}$$

(61) $$\begin{array}{cc} \Lambda_{1} & \;\overset{▵}{=}-\frac{1}{2}\left[\left(\nabla_{A}D_{f}\left(A\right)+\Lambda_{0}A\right)\cdot {\left(\nabla_{A}D_{f}\left(A\right)+\Lambda_{0}A\right)}^{⊤}\right],\;\;\;\;\;\;\;\; \end{array}$$

(62) $$\begin{array}{cc} \Lambda_{2} & \;\overset{▵}{=}-\frac{1}{2}\left[\Lambda_{1}\cdot A\cdot {\nabla_{A}D_{f}\left(A\right)}^{⊤}+\nabla_{A}D_{f}\left(A\right)\cdot {\left(A\right)}^{⊤}\cdot \Lambda_{1}+\Lambda_{0}\cdot \Lambda_{1}+\Lambda_{1}\cdot \Lambda_{0}\right]. \end{array}$$

Additionally, we note that, since finding the global maximum is not guaranteed, it is imperative to initialize $A^{0}$ close to the estimated maximum. In this regard, we leverage the structure of the objective function for each *f*-divergence measure as given in Appendix D. In particular, we observe that the objective of each *f*-divergence measure can be decomposed into two objectives: the first not involving μ (making this objective a convex problem as shown in Section 4), and the second objective a function of μ. Hence, leveraging the structure of the solution from Section 4, we initialize $A^{0}$ such that it maximizes the objective in the case of zero-mean Gaussian models. We further note that, while there are more sophisticated orthogonality constraint-preserving algorithms [45], we find that our method adopted from Reference [43] is sufficient for our purpose, as we show next through numerical simulations.

### 6.2. Results and Discussion

The design of A when μ≠0 is not characterized analytically. Therefore, we resort to numerical simulations to show the gains of optimizing *f*-divergence measures when μ≠0. In particular, we consider the linear discriminant analysis (LDA) problem where the goal is to design a mapping A and perform classification in the lower dimensional space (of dimension *r*). Without loss of generality, we assume n=10 and consider Gaussian densities with the following pre- and post-linear dimensionality reduction structures:

(63) $$\begin{array}{c} \begin{array}{cc} P:\;\; & N\left(0,I_{n}\right)\;\mathrm{and}\;Q:\;\;N\left(\mu ,\Sigma \right)\\ P_{A}:\;\; & N\left(0,I_{r}\right)\;\mathrm{and}\;Q_{A}:\;\;N\left(A\cdot \mu ,h_{1}\left(A\right)\right) \end{array}, \end{array}$$

where the covariance matrix Σ is generated randomly the eigenvalues of which are sampled from a uniform distribution ${\left\{\lambda_{i}∼U\left(0,1\right)\right\}}_{i=1}^{10}$. For the model in (63), we consider two kinds of performance metrics that have information-theoretic performance interpretations: (i) the total probability of error related to the $d_{\mathrm{TV}}\left(A\right)$, and (ii) the exponential decay of error probability related to $D_{\mathrm{KL}}\left(P_{A}\;∥\;Q_{A}\right)$. In what follows, we demonstrate that optimizing appropriate *f*-divergence measures between $P_{A}$ and $Q_{A}$ lead to better performance when compared to the performance of the popular Fisher’s quadratic discriminant analysis (QDA) classifier [20]. In particular, the Fisher’s approach sets r=1 and designs A by solving

(64) $$\begin{array}{c} \underset{A\in R^{1\times n}}{arg max}\;\frac{{\left(\mu \cdot A^{⊤}\right)}^{2}}{A\cdot \left(I_{n}+\Sigma \right)\cdot A^{⊤}}. \end{array}$$

In contrast, we design A such that the information-theoretic objective functions associated with the total probability of error (captured by $d_{\mathrm{TV}}\left(A\right)$) and the exponential decay of error probability (captured by $D_{\mathrm{KL}}\left(P_{A}\;∥\;Q_{A}\right)$) are minimized. The structure of the objective functions is discussed in Total probability of error and Type-II error subjected to type-I error constraints. Both methods and Fisher’s method, after projecting the data into a lower dimension, deploy optimal detectors to discern the true model. It is noteworthy that, in both methods the data in the lower dimensions has a Gaussian model, and the conventional QDA [20] classifier is the optimal detector. Hence, we emphasize that our approach aims to have a design for A that maximizes the distance between the probability measures after reducing the dimensions, i.e., the distance between $P_{A}$ and $Q_{A}$. Since this distance captures the quality of the decisions, our design of A outperforms that of Fisher’s. For each comparison, we consider various values for μ and compare the appropriate performance metrics with that of Fisher’s QDA for each. In all cases, the data is synthetically generated, i.e., sampled from a Gaussian distribution where we consider 2000 data points associated with each measure P and Q.

#### 6.2.1. Schemes for Linear Map

*(1) Total Probability of Error*: In this scheme, the linear map A is designed such that $d_{\mathrm{TV}}\left(A\right)$ is optimized via gradient ascent iterations until convergence. As discussed in Section 4.4.1, since the total probability of error is the key performance metric that arises while optimizing $d_{\mathrm{TV}}\left(A\right)$, it is expected that optimizing $d_{\mathrm{TV}}\left(A\right)$ will result in a smaller total error in comparison to other schemes that optimize other objective functions (e.g., Fisher’s QDA). We note that, since there do not exist closed-form expressions for the total variation distance, we maximize bounds on $d_{\mathrm{TV}}\left(A\right)$ instead via the Hellinger bound in (33) as a proxy to minimize the total probability of error. The corresponding gradient expression to optimize $H^{2}\left(A\right)$ (to perform iterative updates as in (59)) is derived in closed-form and is given in Appendix D.

*(2) Type-II Error Subjected to Type-I Error Constraints*: In this scheme, the linear map A is designed such that $D_{\mathrm{KL}}\left(P_{A}\;∥\;Q_{A}\right)$ is optimized via gradient ascent iterations until convergence. In order to establish a relation, consider the following binary hypothesis test:

(65) $$\begin{array}{c} H_{0}\;:X∼P_{A}\;\mathrm{versus}\;H_{1}\;:X∼Q_{A}. \end{array}$$

When minimizing the probability of type-II error subjected to type-I error constraints, the optimal test guarantees that the probability of type-II error decays exponentially as

(66) $$\begin{array}{c} \lim_{s\to \infty }\frac{-\log(Q_{A}\left(d=H_{0}\right))}{s}=D_{\mathrm{KL}}\left(P_{A}\;∥\;Q_{A}\right), \end{array}$$

where we have define $d:X\to \{H_{0},H_{1}\}$ as the decision rule for the hypothesis test, and *s* denotes the sample size. As a result, $D_{\mathrm{KL}}\left(P_{A}\;∥\;Q_{A}\right)$ appears as the error exponent for hypothesis test in (65). Hence, it is expected that optimizing $D_{\mathrm{KL}}\left(P_{A}\;∥\;Q_{A}\right)$ will result in a smaller type-II error for the same type-I error when comparing with a method that optimizes other objectives (e.g., Fisher’s QDA). The corresponding gradient expression to optimize the $D_{\mathrm{KL}}\left(P_{A}\;∥\;Q_{A}\right)$ is derived in closed-form and is given in Appendix D.

For the sake of comparison and reference, we also consider schemes in which A is designed to optimize the objectives $D_{\mathrm{KL}}\left(A\right)$, the largest eigen modes (LEM), and the smallest eigen modes (SEM), which carry no specific operational significance in the context of the binary classification problem. In the case of LEM and SEM schemes, the linear map A is designed such that the rows of A are the eigenvector associated with the largest and smallest modes of the matrix Σ, respectively. Furthermore, we define 𝟙 as the vector of all those of appropriate dimension.

#### 6.2.2. Performance Comparison

After learning the linear map A for each scheme described in Section 6.2.1, we perform classification in the lower dimensional space of dimension *r* to find the type-I, type-II, and total probability of error for each scheme. Table 1, Table 2, Table 3 and Table 4 tabulate the results for various choices of the mean parameter μ. We have the following important observations: (i) we observe that optimizing $H^{2}\left(A\right)$ results in a smaller total probability of error in comparison to the total error obtained by optimizing the Fisher’s objective; it is important to note that the superior performance is observed despite maximizing bounds on $d_{\mathrm{TV}}\left(A\right)$ (that is sub-optimal) and not the distance itself; and (ii) we observe that except for the case of μ=0.8·𝟙, optimizing $D_{\mathrm{KL}}\left(P_{A}\;∥\;Q_{A}\right)$ results in a smaller type-II error in comparison to that obtained by optimizing the Fisher’s objective indicating a gain in optimizing $D_{\mathrm{KL}}\left(P_{A}\;∥\;Q_{A}\right)$ in comparison to the Fisher’s objective in (64).

It is important to note that the convergence of the gradient ascent algorithm only guarantees a locally optimal solution. While we have restricted the results that consider a maximum separation of μ=0.8·𝟙, we have performed additional simulations for larger separation between models (greater μ>0.8). We have the following observations: (i) solution for the linear map A obtained through gradient ascent becomes highly sensitive to the initialization $A^{0}$; specifically, it was observed that optimizing the Fisher’s objective outperforms optimizing $H^{2}\left(A\right)$ for various initializations $A^{0}$, and vice versa, for other random initializations; and (ii) the gradient ascent solver becomes more prone to getting stuck at the local maxima for larger separations between the models. We conjecture that the odd observation in the case of μ=0.8·𝟙 when optimizing $D_{\mathrm{KL}}\left(P_{A}\;∥\;Q_{A}\right)$ (where optimizing the Fisher’s objective outperforms optimizing $D_{\mathrm{KL}}\left(P_{A}\;∥\;Q_{A}\right)$) supports this observation. Furthermore, we note that, since the problem is convex for μ=0, a deviation from this assumption moves the problem further from being convex, making the solver prone to getting stuck at the locally optimal solutions for larger separation between the Gaussian models.

#### 6.2.3. Subspace Representation

**Figure 6:**
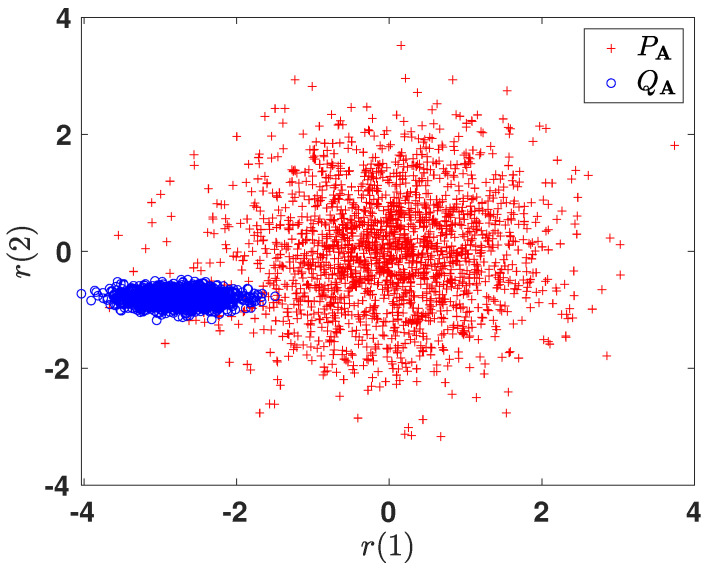
Two-dimensional projected data obtained by optimizing $D_{\mathrm{KL}}\left(P_{A}\;∥\;Q_{A}\right)$.

**Figure 7:**
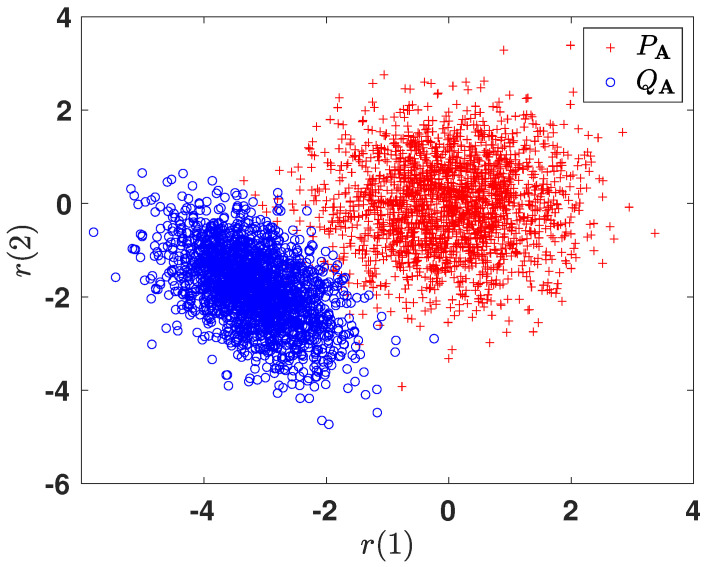
Two-dimensional projected data obtained by optimizing $H^{2}\left(A\right)$.

**Figure 8:**
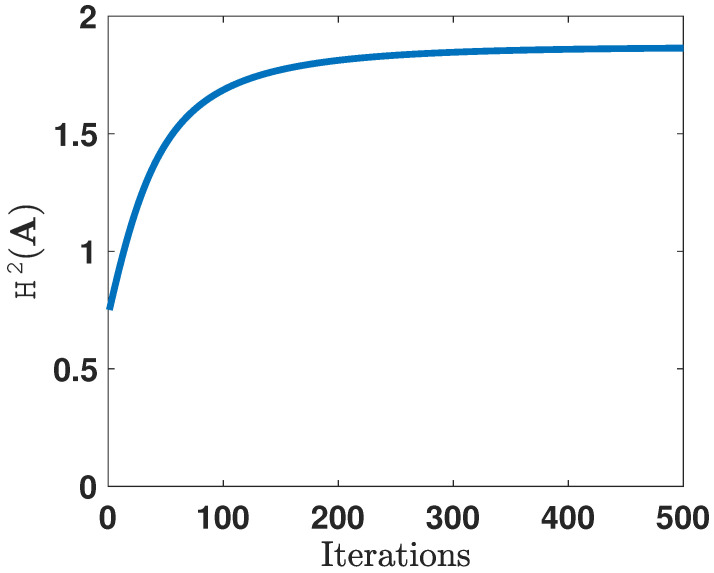
Convergence of the gradient ascent algorithm as a result of optimizing $H^{2}\left(A\right)$.

In order to gain more intuition towards the learned representations, we illustrate the 2-dimensional projections of the original 10-dimensional data obtained after optimizing the corresponding *f*-divergence measures. For brevity, we only show the plots for $D_{\mathrm{KL}}\left(P_{A}\;∥\;Q_{A}\right)$ and $H^{2}\left(A\right)$. Figure 6 and Figure 7 plot the two-dimensional projections of the synthetic dataset that optimize $D_{\mathrm{KL}}\left(P_{A}\;∥\;Q_{A}\right)$ and $H^{2}\left(A\right)$, respectively. As expected, it is observed that the total probability of error is smaller when optimizing $H^{2}\left(A\right)$. Figure 8 shows the variation in the objective function as a function of gradient ascent iterations. As the iterations grow, the objective functions eventually converges to a locally optimal solution.

## 7. Conclusions

In this paper, we have considered the problem of discriminant analysis such that separation between the classes is maximized under *f*-divergence measures. This approach is motivated by dimensionality reduction for inference problems, where we have investigated discriminant analysis under Kullback–Leibler, symmetrized Kullback–Leibler, Hellinger, $\chi^{2}$, and total variation measures. We have characterized the optimal design for the linear transformation of the data onto a lower-dimensional subspace for each in the case of zero-mean Gaussian models and adopted numerical algorithms to find the design of the linear transformation in the case of general Gaussian models with non-zero means. We have shown that, in the case of zero-mean Gaussian models, the row space of the mapping matrix lies in the eigenspace of a matrix associated with the covariance matrix of the Gaussian models involved. While each *f*-divergence measure favors specific eigenvector components, we have shown that all the designs become identical in certain regimes, making the design of the linear mapping independent of the inference problem of interest.

## Figures and Tables

**Table 1:** μ=0.2·𝟙,r=1.

	Fisher’s QDA	$D_{\mathrm{KL}}\left(P_{A}\;∥\;Q_{A}\right)$	$H^{2}\left(A\right)$	$D_{\mathrm{KL}}\left(A\right)$	SEM	LEM
$P_{A}\left(d=H_{1}\right)$	331/2000	331/2000	331/2000	331/2000	337/2000	915/2000
$Q_{A}\left(d=H_{0}\right)$	1226/2000	63/2000	63/2000	63/2000	64/2000	811/2000
Total Error	1557/4000	394/4000	394/4000	394/4000	401/4000	1726/4000

**Table 2:** μ=0.4·𝟙,r=1.

	Fisher’s QDA	$D_{\mathrm{KL}}\left(P_{A}\;∥\;Q_{A}\right)$	$H^{2}\left(A\right)$	$D_{\mathrm{KL}}\left(A\right)$	SEM	LEM
$P_{A}\left(d=H_{1}\right)$	344/2000	344/2000	344/2000	345/2000	347/2000	782/2000
$Q_{A}\left(d=H_{0}\right)$	594/2000	63/2000	63/2000	63/2000	64/2000	739/2000
Total Error	938/4000	407/4000	407/4000	408/4000	411/4000	1521/4000

**Table 3:** μ=0.6·𝟙,r=1.

	Fisher’s QDA	$D_{\mathrm{KL}}\left(P_{A}\;∥\;Q_{A}\right)$	$H^{2}\left(A\right)$	$D_{\mathrm{KL}}\left(A\right)$	SEM	LEM
$P_{A}\left(d=H_{1}\right)$	326/2000	326/2000	335/2000	318/2000	335/2000	638/2000
$Q_{A}\left(d=H_{0}\right)$	137/2000	55/2000	108/2000	57/2000	61/2000	669/2000
Total Error	463/4000	381/4000	443/4000	375/4000	396/4000	1307/4000

**Table 4:** μ=0.8·𝟙,r=1.

	Fisher’s QDA	$D_{\mathrm{KL}}\left(P_{A}\;∥\;Q_{A}\right)$	$H^{2}\left(A\right)$	$D_{\mathrm{KL}}\left(A\right)$	SEM	LEM
$P_{A}\left(d=H_{1}\right)$	264/2000	264/2000	159/2000	255/2000	307/2000	561/2000
$Q_{A}\left(d=H_{0}\right)$	25/2000	53/2000	64/2000	55/2000	60/2000	580/2000
Total Error	289/4000	317/4000	214/4000	310/4000	367/4000	1141/4000

## Data Availability

Not applicable.

## Abbreviations

The following abbreviations are used in this manuscript:

PCA	Principal Component Analysis
MDS	Multidimensional Scaling
SDR	Sufficient Dimension Reduction
DA	Discriminant Analysis
KL	Kullback Leibler
TV	Total Variation
ADD	Average Detection Delay
FAR	False Alarm Rate
CuSum	Cumulative Sum
BC	Bhattacharyya Coefficient
LEM	Largest Eigen Modes
SEM	Smallest Eigen Modes
LDA	Linear Discriminant Analysis
QDA	Quadratic Discriminant Analysis

## Appendix A. Proof of Theorem 1

Consider two pairs of probability measures $\left(P_{A},Q_{A}\right)$ and $\left(P_{\bar{A}},Q_{\bar{A}}\right)$ associated with the mapping A in space X and $\bar{A}$ in space Y, respectively. Let g:X→Y denote any invertible transformation. Under the invertible map, we have

(A1) $$\begin{array}{c} dQ_{\bar{A}}=dQ_{A}\cdot {\left|T\right|}^{-1},\;and\;dP_{\bar{A}}=dP_{A}\cdot {\left|T\right|}^{-1}, \end{array}$$

where |T| denotes the determinant of the Jacobian matrix associated with *g*. Leveraging (A1), the *f*-divergence measure $D_{f}\left(\bar{A}\right)$ simplifies as follows.

(A2) $$\begin{array}{cc} D_{f}\left(\bar{A}\right) & \;\overset{▵}{=}E_{P_{\bar{A}}}\left[f\left(\frac{dQ_{\bar{A}}}{dP_{\bar{A}}}\right)\right]\;\;\;\;\; \end{array}$$

(A3) $$\begin{array}{cc} & =\int_{Y}\;f\left(\frac{dQ_{\bar{A}}}{dP_{\bar{A}}}\right)\;dP_{\bar{A}}\left(y\right) \end{array}$$

(A4) $$\begin{array}{cc} & =\int_{X}{\;|T\left(x\right)|}^{-1}\cdot f\left(\frac{dQ_{A}\cdot {\left|T\left(x\right)\right|}^{-1}}{dP_{A}\cdot {\left|T\left(x\right)\right|}^{-1}}\right)\cdot \left|T\left(x\right)\right|\;dP_{A}\left(x\right) \end{array}$$

(A5) $$\begin{array}{cc} & =\int_{X}\;f\left(\frac{dQ_{A}}{dP_{A}}\right)\;dP_{A}\left(x\right) \end{array}$$

(A6) $$\begin{array}{c} \;=D_{f}\left(A\right).\;\;\;\;\; \end{array}$$

Therefore, *f*-divergence measures are invariant under invertible transformations (both linear and non-linear) ensuring the existence of $\bar{A}$ for every A as a special case for linear transformations.

## Appendix B. Proof of Theorem 3

We observe that $D_{\mathrm{KL}}\left(A\right)$, $D_{\mathrm{SKL}}\left(A\right)$, and the objective to be optimized through the matching bound Section 4.4.2, Matching Bounds up to a Constant on $d_{\mathrm{TV}}\left(A\right)$ can be decomposed as the summation of strictly convex functions involving $g_{\mathrm{KL}}\left(x\right)$, $g_{\mathrm{SKL}}\left(x\right)$, and $g_{\mathrm{TV}}\left(x\right)$, respectively. Since the summation of strictly convex functions is strictly convex, we conclude that each objective $D_{f}\in \left\{D_{\mathrm{KL}}\left(A\right),D_{\mathrm{SKL}}\left(A\right),d_{\mathrm{TV}}\left(A\right)\right\}$ is strictly convex.

Next, the goal is to choose ${\left\{\gamma_{i}\right\}}_{i=1}^{r}$ such that $D_{f}\in \left\{D_{\mathrm{KL}}\left(A\right),D_{\mathrm{SKL}}\left(A\right),d_{\mathrm{TV}}\left(A\right)\right\}$ is maximized subjected to spectral constraints given by $\lambda_{n-(r-i)}\leq \gamma_{i}\leq \lambda_{i}$. In order to choose appropriate $\gamma_{i}$’s, we first note that the global minimizer for functions $g_{f}\in \left\{g_{\mathrm{KL}},g_{\mathrm{SKL}},g_{\mathrm{TV}}\right\}$ appears at x=1. By noting that each $g_{f}$ is strictly convex, it can be readily verified that $g_{f}\left(x\right)$ is monotonically increasing for x>1 and monotonically decreasing for x<1. This will guide selecting ${\left\{\gamma_{i}\right\}}_{i=1}^{r}$, as explained next.

In the case of $\lambda_{n}\geq 1$, i.e., when all the eigenvalues are larger than or equal to 1, the objective of maximizing each $D_{f}\in \left\{D_{\mathrm{KL}}\left(A\right),D_{\mathrm{SKL}}\left(A\right),d_{\mathrm{TV}}\left(A\right)\right\}$ boils down to maximizing a monotonically increasing function (considering the domain). This is trivially done by choosing $\gamma_{i}=\lambda_{i}$ for i∈[r], proving Corollary 1. On the other hand, when $\lambda_{1}\leq 1$, i.e., when all the eigenvalues are smaller than or equal to 1, following the same line of argument, the objective boils down to maximizing each $D_{f}\in \left\{D_{\mathrm{KL}}\left(A\right),D_{\mathrm{SKL}}\left(A\right),d_{\mathrm{TV}}\left(A\right)\right\}$, where each $D_{f}$ is a monotonically decreasing function (considering the domain). This is trivially done by choosing $\gamma_{i}=\lambda_{n-r+i}$ for i∈[r].

When $\lambda_{n}\leq 1\leq \lambda_{1}$, the selection process is not trivial. Rather, an iterative algorithm can be followed, where we start from the eigenvalues farthest away from 1 on both sides and, subsequently, choose the one in every iteration that achieves a higher objective. This procedure can be repeated recursively until *r* eigenvalues are chosen. This procedure is also discussed in Algorithm 1 in Section 4.6.

Finally, constructing the optimal matrix A, which maximizes $D_{f}$ for any data matrix Σ, becomes equivalent to choosing eigenvectors as the rows of A associated with the chosen permutation of eigenvalues for each of the aforementioned cases.

## Appendix C. Proof for Theorem 4

We first find a closed-form expression for $\chi^{2}\left(A\right)$ and $\chi^{2}\left(P_{A}\;∥\;Q_{A}\right)$. From the definition, we have

(A7) $$\begin{array}{cc} \chi^{2}\left(A\right) & \;\overset{▵}{=}\frac{|I_{r}{|}^{\frac{1}{2}}}{{\left(2\pi \right)}^{\frac{r}{2}}\cdot \left|h_{1}\left(A\right)\right|}\cdot \int_{R^{r}}\exp\left[-\frac{1}{2}\cdot \left(Y^{⊤}\cdot K_{1}\cdot Y\right)\right]\;dY-1, \end{array}$$

where we defined $K_{1}\;\overset{▵}{=}2\cdot h_{1}{\left(A\right)}^{-1}-I_{r}$. We note that $K_{1}$ is a real symmetric matrix since $h_{1}\left(A\right)$ is a real symmetric matrix. We denote the eigen decomposition of $K_{1}$ as $K_{1}=U\cdot \Theta \cdot U^{⊤}$, where the matrix Θ is a diagonal matrix with the eigenvalues ${\left\{\theta_{i}\right\}}_{i=1}^{r}$ as its elements. Based on this decomposition, we have

(A8) $$\begin{array}{cc} \chi^{2}\left(A\right) & =\frac{1}{{\left(2\pi \right)}^{\frac{r}{2}}\cdot \left|h_{1}\left(A\right)\right|}\cdot \int_{R^{r}}\exp\left[-\frac{1}{2}\left(Y^{⊤}\cdot U\Theta U^{⊤}\cdot Y\right)\right]\;dY-1\; \end{array}$$

(A9) $$\begin{array}{cc} & =\frac{1}{{\left(2\pi \right)}^{\frac{r}{2}}\cdot \left|h_{1}\left(A\right)\right|}\cdot \int_{R^{r}}\exp\left[-\frac{1}{2}\left(W^{⊤}\cdot \Theta \cdot W\right)\right]\;dW-1 \end{array}$$

(A10) $$\begin{array}{cc} & =\frac{1}{{\left(2\pi \right)}^{\frac{r}{2}}\cdot \left|h_{1}\left(A\right)\right|}\cdot \prod_{i=1}^{r}\int_{-\infty }^{\infty }\exp\left[-\frac{1}{2}\left(\theta_{i}\cdot w_{i}^{2}\right)\right]\;dw_{i}-1, \end{array}$$

where we have defined $W\;\overset{▵}{=}U^{⊤}\cdot Y$. We note that, in order for $\chi^{2}\left(A\right)$ to be finite, it is required that the eigenvalues ${\left\{\theta_{i}\right\}}_{i=1}^{r}$ be non-negative. Hence, based on the definition of $K_{1}$, all the eigenvalues $\lambda_{i}$ should fall in the interval (0,2). Hence, we obtain:

(A11) $$\begin{array}{cc} \chi^{2}\left(A\right) & =\frac{1}{{\left(2\pi \right)}^{\frac{r}{2}}\cdot \left|h_{1}\left(A\right)\right|}\cdot \prod_{i=1}^{r}\int_{-\infty }^{\infty }\exp\left[-\frac{1}{2}\left(\theta_{i}\cdot w_{i}^{2}\right)\right]\;dw_{i}-1 \end{array}$$

(A12) $$\begin{array}{cc} & =\frac{1}{{\left(2\pi \right)}^{\frac{r}{2}}\cdot \left|h_{1}\left(A\right)\right|}\cdot \prod_{i=1}^{r}\sqrt{\frac{2\pi }{\theta_{i}}}-1\;\;\;\;\;\; \end{array}$$

(A13) $$\begin{array}{cc} & =\frac{1}{|h_{1}\left(A\right)|}\cdot \sqrt{\frac{1}{|K_{1}|}}-1.\;\;\;\;\;\;\;\;\; \end{array}$$

Recall that the eigenvalues of $h_{1}\left(A\right)$ are given by ${\left\{\gamma_{i}\right\}}_{i=1}^{r}$ in the descending order. Therefore, (A13) simplifies to:

(A14) $$\begin{array}{c} \chi^{2}\left(A\right)=\prod_{i=1}^{r}\sqrt{\frac{1}{\gamma_{i}\cdot \left(2-\gamma_{i}\right)}}-1=\prod_{i=1}^{r}g_{\chi_{1}}\left(\gamma_{i}\right)-1. \end{array}$$

Hence, from (A14), maximizing $\chi^{2}\left(A\right)$ is equivalent to choosing the eigenvalues ${\left\{\gamma_{i}\right\}}_{i=1}^{r}$ such that they maximize $g_{\chi_{1}}\left(x\right)$. Similarly, the closed-form expression for $\chi^{2}\left(P_{A}\;∥\;Q_{A}\right)$ can be derived as follows:

(A15) $$\begin{array}{cc} \chi^{2}\left(P_{A}\;∥\;Q_{A}\right) & =\frac{|h_{1}{\left(A\right)|}^{\frac{1}{2}}}{{\left(2\pi \right)}^{\frac{r}{2}}\cdot \left|I_{r}\right|}\cdot \int_{R^{r}}\exp\left[-\frac{1}{2}\cdot \left(Y^{⊤}\cdot K_{2}\cdot Y\right)\right]\;dY-1, \end{array}$$

where we defined $K_{2}\;\overset{▵}{=}2\cdot I_{r}-h_{1}{\left(A\right)}^{-1}$. We note that $K_{2}$ is a real symmetric matrix due to $h_{1}\left(A\right)$ being a real symmetric matrix. Hence, following a similar line of argument as in the case of $\chi^{2}\left(A\right)$, and as a consequence of Theorem 2, we conclude that all the eigenvalues $\lambda_{i}$ should fall in the interval (0.5,∞) to ensure a finite value for $\chi^{2}\left(P_{A}\;∥\;Q_{A}\right)$. Following this requirement, since the integrands are bounded, we obtain the following closed-form expression:

(A16) $$\begin{array}{c} \chi^{2}\left(P_{A}\;∥\;Q_{A}\right)=\frac{|h_{1}\left(A\right){|}^{\frac{1}{2}}}{1}\cdot \sqrt{\frac{1}{|K_{2}|}}-1. \end{array}$$

Recall that the eigenvalues of $h_{1}\left(A\right)$ are given by ${\left\{\gamma_{i}\right\}}_{i=1}^{r}$; then, (A16) simplifies to

(A17) $$\begin{array}{c} \chi^{2}\left(P_{A}\;∥\;Q_{A}\right)=\prod_{i=1}^{r}\sqrt{\frac{\gamma_{i}^{2}}{\left(2\gamma_{i}-1\right)}}-1=\prod_{i=1}^{r}g_{\chi_{2}}\left(\gamma_{i}\right)-1. \end{array}$$

Hence, from (A17), maximizing $\chi^{2}\left(P_{A}\;∥\;Q_{A}\right)$ is equivalent to choosing the eigenvalues ${\left\{\gamma_{i}\right\}}_{i=1}^{r}$ such that they maximize $g_{\chi_{2}}\left(x\right)$.

We observe that $H^{2}\left(A\right)$, $\chi^{2}\left(A\right)$, and $\chi^{2}\left(P_{A}\;∥\;Q_{A}\right)$ can be decomposed as the product of *r* non-negative identical convex functions involving $g_{H}\left(x\right)$, $g_{\chi_{1}}\left(x\right)$, and $g_{\chi_{2}}\left(x\right)$, respectively. Hence, the goal is to choose ${\left\{\gamma_{i}\right\}}_{i=1}^{r}$ such that $D_{f}\in \left\{H^{2}\left(A\right),\chi^{2}\left(A\right),\chi^{2}\left(P_{A}\;∥\;Q_{A}\right)\right\}$ is maximized subjected to spectral constraints given by $\lambda_{n-(r-i)}\leq \gamma_{i}\leq \lambda_{i}$. In order to choose appropriate $\gamma_{i}$’s, we first note that the global minimizer for each $g_{f}\in \left\{g_{H},g_{\chi_{1}},g_{\chi_{2}}\right\}$ is attained at x=1. Leveraging this observation, along with the structure that each $g_{f}$ is convex, it is easy to infer that each $g_{f}\left(x\right)$ is monotonically increasing for x>1 and monotonically decreasing x<1. From the exact same argument in Appendix B, we obtain Corollaries 3 and 4.

Therefore, similar to Appendix B, constructing the linear map A that maximizes $D_{f}\in \left\{H^{2}\left(A\right),\chi^{2}\left(A\right),\chi^{2}\left(P_{A}\;∥\;Q_{A}\right)\right\}$ for any data matrix Σ boils down to choosing eigenvectors as rows of A associated with the chosen permutation of eigenvalues for each of the aforementioned cases.

## Appendix D. Gradient Expressions for f-Divergence Measures

For clarity in analysis, we define the following functions:

(A18) $$\begin{array}{cc} h_{2}\left(A\right) & \;\overset{▵}{=}\mu^{⊤}\cdot A^{⊤}\cdot A\cdot \mu ,\;\;\;\; \end{array}$$

(A19) $$\begin{array}{cc} h_{3}\left(A\right) & \;\overset{▵}{=}\mu^{⊤}\cdot A^{⊤}\cdot {\left[h_{1}\left(A\right)\right]}^{-1}\cdot A\cdot \mu . \end{array}$$

Based on these definitions, we have the following representations for the divergence measures and their associated gradients:

(A20) $$\begin{array}{cc} D_{\mathrm{KL}}\left(A\right) & =\frac{1}{2}\left[\log\frac{1}{|h_{1}\left(A\right)|}-r+\mathrm{Tr}\left[h_{1}\left(A\right)\right]+h_{2}\left(A\right)\right],\\ \nabla_{A}D_{\mathrm{KL}}\left(A\right) & ={\left[h_{1}\left(A\right)\right]}^{-1}\cdot \left[I_{r}-{\left[h_{1}\left(A\right)\right]}^{-1}-A\cdot \mu \cdot \mu^{⊤}\cdot A^{⊤}\cdot {\left[h_{1}\left(A\right)\right]}^{-1}\right]\cdot A\cdot \Sigma \\ & \;+{\left[h_{1}\left(A\right)\right]}^{-1}\cdot A\cdot \mu \cdot \mu^{⊤}. \end{array}$$

(A21) $$\begin{array}{cc} D_{\mathrm{KL}}\left(P_{A}\;∥\;Q_{A}\right) & =\frac{1}{2}\left[\log|h_{1}\left(A\right)|-r+\mathrm{Tr}\left[h_{1}{\left(A\right)}^{-1}\right]+h_{3}\left(A\right)\right],\\ \nabla_{A}D_{\mathrm{KL}}\left(P_{A}\;∥\;Q_{A}\right) & =\left(I_{r}-{\left[h_{1}\left(A\right)\right]}^{-1}\right)\cdot A\cdot \Sigma +A\cdot \mu \cdot \mu^{⊤}. \end{array}$$

(A22) $$\begin{array}{cc} D_{\mathrm{SKL}}\left(A\right) & =\frac{1}{2}\cdot \left[\mathrm{Tr}\left({\left[h_{1}\left(A\right)\right]}^{-1}+h_{1}\left(A\right)\right)+h_{2}\left(A\right)+h_{3}\left(A\right)\right]-r,\\ \nabla_{A}D_{\mathrm{SKL}}\left(A\right) & =\left[I_{r}-{\left[h_{1}\left(A\right)\right]}^{-2}-{\left[h_{1}\left(A\right)\right]}^{-1}\cdot A\cdot \mu \cdot \mu^{⊤}\cdot A^{⊤}\cdot {\left[h_{1}\left(A\right)\right]}^{-1}\right]\cdot A\cdot \Sigma \end{array}$$

(A23) $$\begin{array}{cc} & \;+\left(I_{r}+{\left[h_{1}\left(A\right)\right]}^{-1}\right)\cdot A\cdot \mu \cdot \mu^{⊤}.\;\;\;\;\;\;\;\; \end{array}$$

(A24) $$\begin{array}{cc} H^{2}\left(A\right) & =2-2\frac{|4\cdot h_{1}{\left(A\right)|}^{\frac{1}{4}}}{|h_{1}\left(A\right)+I_{r}{|}^{\frac{1}{2}}}\cdot \exp\left(-\frac{\mu^{⊤}\cdot A^{⊤}\cdot {\left[h_{1}\left(A\right)+I_{r}\right]}^{-1}\cdot A\cdot \mu }{4}\right),\\ \frac{\nabla_{A}H^{2}\left(A\right)}{-[1-H^{2}\left(A\right)]} & =\frac{1}{2}\cdot {\left[h_{1}\left(A\right)\right]}^{-1}\cdot A\cdot \Sigma +{\left[h_{1}\left(A\right)+I_{r}\right]}^{-1}\cdot \left[-A\cdot \left[\Sigma +I_{n}\right]-\frac{1}{2}\cdot A\cdot \mu \cdot \mu^{⊤}\right.\\ & \;+\left.\frac{1}{2}\cdot A\cdot \mu \cdot \mu^{⊤}\cdot A^{⊤}\cdot {\left[h_{1}\left(A\right)+I_{r}\right]}^{-1}\cdot A\cdot \left[\Sigma +I_{n}\right]\right]. \end{array}$$

## Appendix E. Proof for Lagrange Multipliers

Denoting $\nabla_{A}L$ by $\tilde{\Delta }$ and $\nabla_{A}D_{f}$ by Δ, and further post-multiplying (59) by $A^{k+1}$, we have:

(A25) $$\begin{array}{cc} A^{k+1}\cdot {\left(A^{k+1}\right)}^{⊤} & =A^{k}\cdot {\left(A^{k+1}\right)}^{⊤}+\alpha \cdot \tilde{\Delta }\cdot {\left(A^{k+1}\right)}^{⊤},\;\;\;\;\;\;\;\; \end{array}$$

(A26) $$\begin{array}{cc} \;\;I_{r} & =A^{k}\cdot {\left(A^{k}+\alpha \cdot \tilde{\Delta }\right)}^{⊤}+\alpha \cdot \tilde{\Delta }\cdot {\left(A^{k}+\alpha \cdot \tilde{\Delta }\right)}^{⊤}, \end{array}$$

(A27) $$\begin{array}{cc} 0 & =A^{k}\cdot {\tilde{\Delta }}^{⊤}+\tilde{\Delta }\cdot {\left(A^{k}\right)}^{⊤}+\alpha \cdot \tilde{\Delta }\cdot {\tilde{\Delta }}^{⊤}.\;\; \end{array}$$

Substituting $\tilde{\Delta }=\Delta +\Lambda \cdot A$ in (A27) and simplifying the expression, we obtain:

(A28) $$\begin{array}{cc} 2\cdot \Lambda +A^{k}\cdot \Delta^{⊤}+\Delta \cdot {\left(A^{k}\right)}^{⊤} & =-\alpha \cdot (\Delta \cdot \Delta^{⊤}+\Delta \cdot {\left(A^{k}\right)}^{⊤}\Lambda +\Lambda \cdot A^{k}\cdot \Delta^{⊤}+\Lambda \cdot \Lambda^{⊤}). \end{array}$$

By noting that Λ is symmetric, taking the Taylor series expansions of Λ around α=0 and equating the terms of α in both sides, we obtain the relationships in (60)–(62).

## References

[1] Kunisky D., Wein A.S., Bandeira A.S. (2019). Notes on computational hardness of hypothesis testing: Predictions using the low-degree likelihood ratio. arXiv.

[2] Gamarnik D., Jagannath A., Wein A.S. (2020). Low-degree hardness of random optimization problems. arXiv.

[3] van der Maaten L., Postma E., van den Herik J. (2009). Dimensionality reduction: A comparative review. J. Mach. Learn. Res..

[4] Lee J.A., Verleysen M. (2007). Nonlinear Dimensionality Reduction.

[5] DeMers D., Cottrell G.W. Non-linear dimensionality reduction. Proceedings of the Advances in Neural Information Processing Systems.

[6] Cunningham J.P., Ghahramani Z. (2015). Linear dimensionality reduction: Survey, insights, and generalizations. J. Mach. Learn. Res..

[7] Pearson K. (1901). On lines and planes of closest fit to systems of points in space. Philos. Mag..

[8] Eckart C., Young G. (1936). The approximation of one matrix by another of lower rank. Psychometrika.

[9] Jolliffe I. (2002). Principal Component Analysis.

[10] Torgerson W.S. (1952). Multidimensional scaling: I. Theory and method. Psychometrika.

[11] Cox T.F., Cox M.A. (2008). Multidimensional scaling. Handbook of Data Visualization.

[12] Borg I., Groenen P.J. (2005). Modern Multidimensional Scaling: Theory and Applications.

[13] Izenman A.J. (2013). Linear discriminant analysis. Modern Multivariate Statistical Techniques.

[14] Globerson A., Tishby N. (2003). Sufficient dimensionality reduction. J. Mach. Learn. Res..

[15] Fisher R.A. (1936). The use of multiple measurements in taxonomic problems. Ann. Eugen..

[16] Rao C.R. (1948). The utilization of multiple measurements in problems of biological classification. J. R. Stat. Soc. Ser. B.

[17] Fukunaga K. (2013). Introduction to Statistical Pattern Recognition.

[18] Suresh B., Ganapathiraju A. (1998). Linear discriminant analysis- A brief tutorial. Inst. Signal Inf. Process..

[19] Bishop C.M. (2006). Pattern Recognition and Machine Learning.

[20] Hastie T., Tibshirani R., Friedman J. (2009). The Elements of Statistical Learning: Data Mining, Inference, and Prediction.

[21] Shannon C.E. (1948). A mathematical theory of communication. Bell Syst. Tech. J..

[22] Kullback S., Leibler R.A. (1951). On information and sufficiency. Ann. Math. Stat..

[23] Gelfand I.M., Kolmogorov A.N., Yaglom A.M. (1956). On the general definition of the amount of information. Dokl. Akad. Nauk SSSR.

[24] Csiszár I. (1948). Eine Informationstheoretische Ungleichung und ihre Anwendung auf den Bewis der Ergodizität von Markhoffschen Ketten. Magy. Tudományos Akad. Mat. Kut. Intézetének Közleményei.

[25] Ali S.M., Silvey S.D. (1966). General Class of Coefficients of Divergence of One Distribution from Another. J. R. Stat. Soc..

[26] Morimoto T. (1963). Markov Processes and the H-Theorem. J. Phys. Soc. Jpn..

[27] Arimoto S. (1971). Information-theoretical considerations on estimation problems. Inf. Control.

[28] Barron A.R., Gyorfi L., Meulen E.C. (1992). Distribution estimation consistent in total variation and in two types of information divergence. IEEE Trans. Inf. Theory.

[29] Berlinet A., Vajda I., Meulen E.C. (1998). About the asymptotic accuracy of Barron density estimates. IEEE Trans. Inf. Theory.

[30] Gyorfi L., Morvai G., Vajda I. Information-theoretic methods in testing the goodness of fit. Proceedings of the IEEE International Symposium on Information Theory.

[31] Liese F., Vajda I. (2006). On Divergences and Informations in Statistics and Information Theory. IEEE Trans. Inf. Theory.

[32] Kailath T. (1967). The Divergence and Bhattacharyya Distance Measures in Signal Selection. IEEE Trans. Commun. Technol..

[33] Poor H. (1980). Robust decision design using a distance criterion. IEEE Trans. Inf. Theory.

[34] Clarke B.S., Barron A.R. (1990). Information-theoretic asymptotics of Bayes methods. IEEE Trans. Inf. Theory.

[35] Harremoes P., Vajda I. (2011). On Pairs of *f*-divergences and their joint range. IEEE Trans. Inf. Theory.

[36] Sason I., Verdú S. (2016). *f*-Divergence Inequalities. IEEE Trans. Inf. Theory.

[37] Sason I. (2018). On *f*-divergence: Integral representations, local behavior, and inequalities. Entropy.

[38] Rao C.R., Statistiker M. (1973). Linear Statistical Inference and Its Applications.

[39] Poor H.V., Hadjiliadis O. (2008). Quickest Detection.

[40] Cavanaugh J.E. (2004). Criteria for linear model selection based on Kullback’s symmetric divergence. Aust. N. Z. J. Stat..

[41] Devroye L., Mehrabian A., Reddad T. (2020). The total variation distance between high-dimensional Gaussians. arXiv.

[42] Pollak M. (1985). Optimal detection of a change in distribution. Ann. Stat..

[43] Carter K.M., Raich R., Finn W.G., Hero A.O. (2009). Information preserving component analysis: Data projections for flow cytometry analysis. IEEE J. Sel. Top. Signal Process..

[44] Wen Z., Yin W. (2013). A feasible method for optimization with orthogonality constraints. Math. Program..

[45] Edelman A., Arias T., Smith S. (1998). The geometry of algorithms with orthogonality constraints. SIAM J. Matrix Anal. Appl..

